# Brain Acetyl-CoA Production and Phosphorylation of Cytoskeletal Proteins Are Targets of CYP46A1 Activity Modulation and Altered Sterol Flux

**DOI:** 10.1007/s13311-021-01079-6

**Published:** 2021-07-07

**Authors:** Natalia Mast, Alexey M. Petrov, Erin Prendergast, Ilya Bederman, Irina A. Pikuleva

**Affiliations:** 1 Department of Ophthalmology and Visual Sciences, Case Western Reserve University, Cleveland, OH USA; 2 Laboratory of Biophysics of Synaptic Processes, Kazan Institute of Biochemistry and Biophysics, Federal Research Center, Kazan Scientific Center of RAS”, 2/31 Lobachevsky Street, Box 30, 420111 Kazan, Russia; 3 Institute of Neuroscience, Kazan State Medial University, 49 Butlerova Street, 420012 Kazan, Russia; 4 Department of Genetics and Genome Sciences, Case Western Reserve University, Cleveland, OH USA

**Keywords:** CYP46A1, Acetyl-CoA, Cytoskeleton, Phosphorylation, Sterol flux, Alzheimer’s disease

## Abstract

Cholesterol and 24-hydroxycholesterol are the most abundant brain sterols and represent the substrate and product, respectively, of cytochrome P450 46A1 (CYP46A1), a CNS-specific enzyme. CYP46A1 controls cholesterol elimination and turnover in the brain, the two processes that determine the rate of brain sterol flux through the plasma membranes and thereby the properties of these membranes. Brain sterol flux is decreased in *Cyp46a1*^*−/−*^ mice compared to wild-type mice and increased in 5XFAD mice (a model of Alzheimer’s disease) when they are treated with a small dose of efavirenz, a CYP46A1 activator. Herein, we first assessed the brain proteome (synaptosomal fractions) and phospho-proteome (synaptosomal fractions and brain homogenates) of efavirenz-treated and control 5XFAD mice. Then, based on the pattern of protein abundance change, we conducted acetyl-CoA measurements (brain homogenates and mitochondria) and metabolic profiling (brain homogenates). The phospho-proteomics datasets were used for comparative analyses with the datasets obtained by us previously on mice with the same changes (efavirenz-treated and control 5XFAD mice from a different treatment paradigm) or with changes in the opposite direction (*Cyp46a1*^*−/−*^ vs wild-type mice) in brain sterol flux. We found that CYP46A1 activity or the rate of brain sterol flux affects acetyl-CoA-related metabolic pathways as well as phosphorylation of cytoskeletal and other proteins. Knowledge of the key roles of acetyl-CoA and cytoskeletal phosphorylation in cell biology expands our understanding of the significance of CYP46A1-mediated cholesterol 24-hydroxylation in the brain and provides an additional explanation for why CYP46A1 activity modulations are beneficial in mouse models of different brain diseases.

**Supplementary Information:**

The online version contains supplementary material available at 10.1007/s13311-021-01079-6.

Cytochrome P450 46A1 (CYP46A1) plays a key role in cholesterol homeostasis in the brain by converting cholesterol to 24-hydroxycholesterol [1, 2]. This reaction removes the majority of excess brain cholesterol because unlike cholesterol, 24-hydroxycholesterol can enter the systemic circulation and reach the liver, where it is further metabolized to bile acids [1, 3]. Animal studies have demonstrated that increases in CYP46A1 activity by genetic or pharmacologic means can be beneficial in models of Alzheimer’s and Huntington’s diseases, Niemann-Pick disease type C, spinocerebellar ataxia, depression, glioblastoma and prion disease [4–14]. A clinical trial is in progress to evaluate the effects of efavirenz (EFV), a CYP46A1 activator, on people with mild cognitive impairment due to Alzheimer’s disease (ClinicalTrials.gov: NCT03706885). CYP46A1 inhibition can also be beneficial and ameliorate neuronal hyperexcitation in mouse models of seizures and epilepsy [15]. A clinical trial of children with frequent seizures who received soticlestat, a CYP46A1 inhibitor (ClinicalTrials.gov: NCT03650452), was recently completed. No results have been published yet, but there is a press release (https://ovidrx.com/science/clinical-studies/) stating that the study achieved its primary endpoint—a reduction in seizure frequency from the baseline.

This laboratory discovered CYP46A1 activation by pharmacologic means, namely, by EFV, and tested two paradigms of EFV treatment in 5XFAD mice, a model of Alzheimer’s disease [7, 13, 16, 17]. In both paradigms (Fig. 1), the same small drug dose was used (0.1 mg/kg of body weight/day), and animals were treated until the same age of 9 months, when the amyloid β load started to plateau in the 5XFAD brain [17]. However, the beginning and duration of treatment were different: from 1 month of age (and hence for 8 months) in the first treatment paradigm (1TP) and from 3 months of age (and hence for 6 months) in the second treatment paradigm (2TP). The 2TP was more clinically relevant than the 1TP, as the 2TP was conducted on mature animals and started when mice already had amyloid plaques in the brain [7]. Conversely, in the 1TP, EFV administration began when animals were still young and had not yet developed amyloid plaques in the brain. However, both treatments activated CYP46A1, enhanced brain cholesterol turnover, improved mouse performance in behavioural tests and had differential effects on the amyloid β load and expression of synaptic proteins as well as markers of inflammation [7, 13]. In addition, 5XFAD mice from the 1TP were characterized for the EFV effect on the brain phospho-proteome, which was found to be altered [18].

**Fig. 1:**
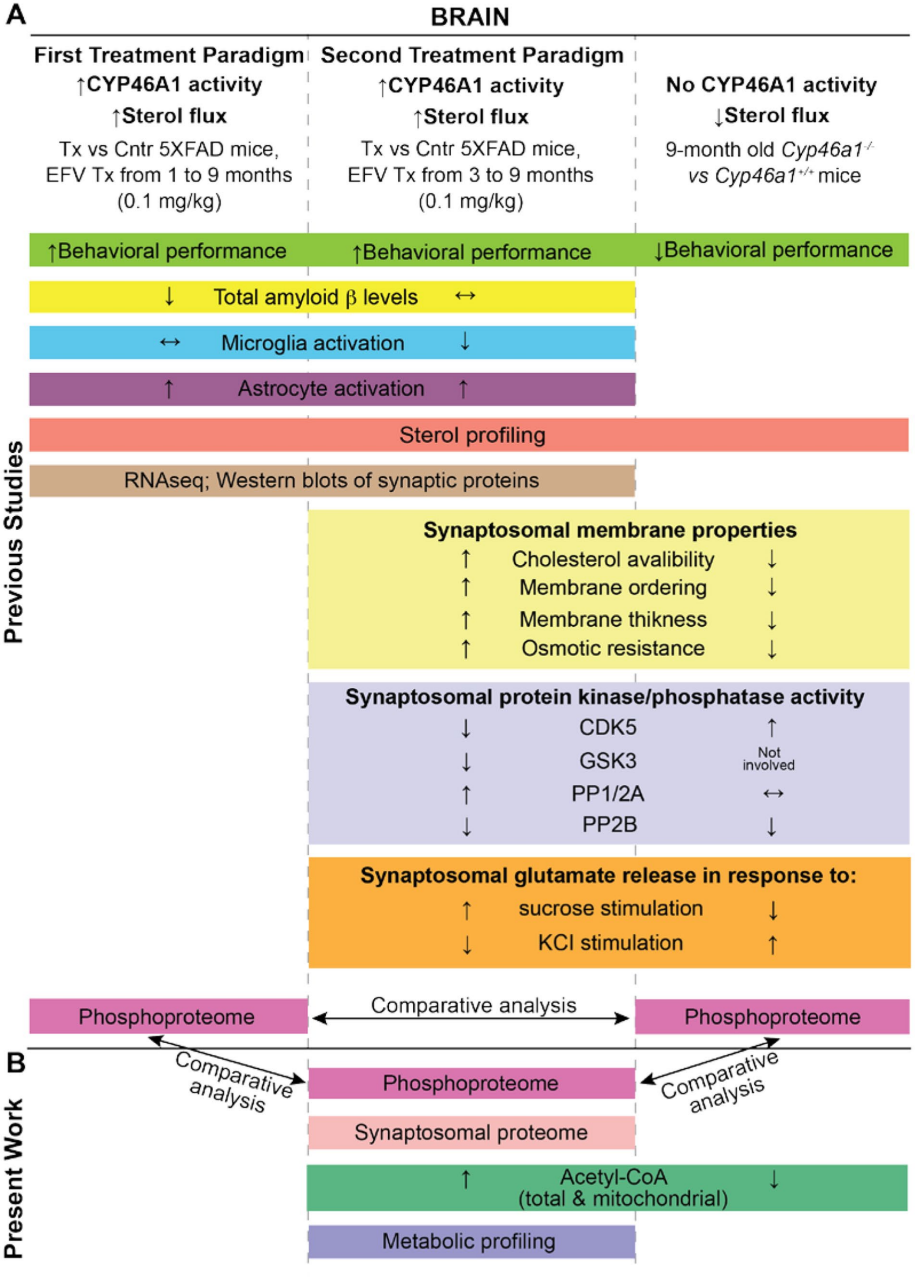
Study design. **A** Summary of some of the previously published studies of mice with increased (↑) CYP46A1 activity and brain sterol flux (EFV-treated (Tx) vs control (Cntr) 5XFAD mice) and animals with no CYP46A1 activity and decreased (↓) sterol flux (*Cyp46a1*^*−/−*^ vs wild-type mice) [7, 13, 18, 20, 22, 50, 92]. **B** Summary of experiments and comparative analyses conducted in the present work. Similar types of experiments are placed in boxes of the same colour. See “Introduction” for details

A comparison of pharmacologic and genetic modulations of CYP46A1 activity in mouse models of different diseases revealed that several apparently unlinked biological processes were affected [4–6, 9–13, 19, 20]. Therefore, we introduced a unifying sterol flux hypothesis to explain the diversity of the observed effects. According to this hypothesis, altered cholesterol turnover and overall sterol flux through the plasma membranes due to CYP46A1 activity modulation affect the membrane physico-chemical properties and thereby membrane-bound proteins and membrane-dependent events [18]. In particular, altered sterol flux can change the activity and targeting of membrane-associated protein kinases or protein phosphatases to their protein substrates and vice versa and thereby modulate the extent of protein phosphorylation [18].

We began testing our hypothesis and characterized synaptosomal fractions from the brains of *Cyp46a1*^*−/−*^ and wild-type animals as well as EFV-treated and vehicle-treated (control) 5XFAD mice from the 2TP [20]. These two groups of animals (Fig. 1), which had opposite changes in the rate of brain sterol flux, also had opposite changes in membrane ordering, thickness, resistance to osmotic stress, cholesterol availability and exocytotic glutamate release [20]. In addition, the activity of several enzymes involved in the regulation of exocytotic glutamate release appeared to be altered in mouse synaptosomal fractions, with a common decrease in the activity of CDK5 (cyclin-dependent kinase 5; all protein abbreviations are according to UniProt [21]) and PP2B (protein phosphatase 2B) and group-specific changes in the activity of GSK3 (glycogen synthase kinase 3) and PP1/PP2A (protein phosphatase 1/protein phosphatase 2A) [20]. This result was consistent with decreases in the activity of CDK5 and GSK3 predicted computationally based on the changes in the brain phospho-proteome of *Cyp46a1*^*−/−*^ vs wild-type mice and EFV-treated vs control 5XFAD mice from the 1TP [18, 22]. Herein, we continued to further assess the significance of CYP46A1 or brain sterol flux and test our hypothesis. We conducted additional characterizations of mice with increased CYP46A1 activity and brain sterol flux (EFV-treated vs control 5XFAD mice from the 2TP) as well as animals with no CYP46A1 activity and decreased brain sterol flux (*Cyp46a1*^*−/−*^ vs wild-type mice). These characterizations provided new and unexpected mechanistic insights and enabled comparisons with the datasets obtained in our previous studies (Fig. 1). We obtained additional support for the sterol flux hypothesis and expanded our understanding of how CYP46A1 can be a common therapeutic target for various brain disorders.

## Material and Methods

### Animals

Studies were conducted on 5XFAD mice (The Jackson Laboratory), which are transgenic animals hemizygous for the mutant (K670N, M671L, I716V, V717I) human amyloid precursor protein 695 and mutant (M146L and L286V) human presenilin 1 [17]. 5XFAD^*Tg/0*^ males were crossed with wild-type B6SJL females, and only the F1 generation of hemizygous animals was then used. EFV administration followed the 2TP, namely, the drug was given to mice from 3 to 9 months of age at a dose of 0.1 mg/kg body weight/day delivered in drinking water containing 0.0004% Tween 80 [13]. Control animals received vehicle (aqueous 0.0004% Tween 80). Mice in the present work were from the same cohort of animals, which was characterized previously and shown to have increased cholesterol turnover and sterol flux [20]. For the measurements of the acetyl coenzyme A (acetyl-CoA) levels, we also used sex- and age-matched B6SJL mice, a background strain for 5XFAD mice, as well as *Cyp46a1*^*−/−*^ and wild-type mice on the C57BL/6 J;129S6/SvEv background, which were generated as described [7, 22]. All mice were maintained in a temperature- and humidity-controlled environment with a 12-h light–dark cycle with standard rodent chow and water provided ad libitum. All animal experiments were approved by the Institutional Animal Care and Use Committee and conformed to recommendations of the American Veterinary Association Panel on Euthanasia. Only male mice were used, as there were no sex differences in EFV treatment effects on the brain sterol profile and animal performance in behavioural tasks [13, 20]. However, we acknowledge that women are known to be more vulnerable to Alzheimer’s disease than men and that humans have sex differences in terms of the hallmarks and other manifestations of this disease [23, 24]. Mice were selected from the pool of all available animals and randomly assigned to either the control or treatment group, which were matched by size, age (3 months old) and sex. Sample size was based on our previous experience. Experimenters were not blinded with respect to mouse genotype or EFV treatment.

### Brain Processing

Brain isolation was followed by removal of the cerebellum and brainstem. The remaining tissue was rinsed in cold phosphate-buffered saline and blotted. Either the whole brain or one hemisphere was then used to prepare brain homogenates.

### Brain Phospho-Proteome

Brain homogenates were prepared from one hemisphere (four biological replicates per group) and processed as described [18, 20, 22]. The same facility, the Proteomics Core at the Cleveland Clinic Foundation, conducted these and our previous characterizations of the wild-type and *Cyp46a1*^*−/−*^ mice as well as of the control and EFV-treated 5XFAD mice from the 1TP [18, 22].

### Proteome and Phospho-Proteome of Synaptosomal Fractions

Synaptosomal fractions were prepared as described [20] from the whole brain using 5 biological replicates per group for EFV-treated 5XFAD mice and 3 biological replicates per group for control 5XFAD mice. Samples were analysed by Bioproximity, LLC (Manassas, VA, USA), a proteomics mass spectrometry (MS) company (https://www.bioproximity.com/). Briefly, cerebellum- and brainstem-free brains were homogenized on ice in a Dounce homogenizer (14–15 strokes per sample) in Syn-PER™ Synaptic Protein Extraction Reagent (Thermo Fisher Scientific #87,793) to prepare 10% homogenates. Homogenates were then subjected to differential centrifugation, first at 1200 g and 4 °C for 10 min and then at 14,000 g and 4 °C for 30 min. The pellet after the second centrifugation was resuspended in 0.5 ml of Syn-PER™ reagent, aliquoted, flash frozen in liquid nitrogen and stored until use at −80 °C. For omics analyses, proteins were extracted from synaptosomal fractions with trifluoroacetic acid [25], precipitated with acetone [26] and reconstituted in 1% SDS containing 50 mM Tris–HCl, pH 8.0, 5 mM Tris(2‐carboxyethyl)phosphine hydrochloride and 20 mM chloroacetamide. Solutions were heated to 95 °C for 10 min, cooled, probe-sonicated and centrifuged for clarification. Proteins were digested with trypsin using the SP3 method [27]. Phosphopeptides were enriched from peptide digests using Fe-NTA magnetic agarose beads (Cube Biotech) [28]. Digestion mixtures were analysed by ultra-high-performance liquid chromatography tandem mass spectrometry (UHPLC-MS/MS). Liquid chromatography (LC) was carried out on an Easy-nLC 1200 UHPLC system (Thermo Fisher Scientific) with mobile phase A being 99.9% Milli-Q water containing 0.1% formic acid and mobile phase B being 80% acetonitrile containing 0.1% formic acid. The 60 min LC gradient ran from 0% B to 25% B over 50 min, ran to 80% B over 1 min and held at 80% B for the remaining 9 min. Samples were loaded directly onto the column (15 cm × 100 μm inner diameter packed with 1.9 micron ReproSil-Pur C18 media (Dr. Maisch)). The LC was interfaced to a quadrupole-Orbitrap mass spectrometer (Q Exactive HF-X, Thermo Fisher Scientific) via nano-electrospray ionization. An electrospray voltage of 2.2 kV was applied. The mass spectrometer acquired tandem mass spectra from the top 12 ions in the full scan from 350 to 1400 m/z by data-dependent acquisition. Dynamic exclusion was set to 30 s, singly charged ions were excluded and the isolation width was set to 1.6 Da, with full MS resolution of 60,000 and MS/MS resolution of 15,000. The normalized collision energy was set to 27, automatic gain control to 3e6, max fill MS to 45 ms and max fill MS/MS to 22 ms.

Mass spectrometer RAW data files were converted to mzML format using msconvert [29]. MGF files were generated using OpenMS [30]. All searches were performed on Amazon Web Services–based compute instances. Detailed search parameters are printed in the search output XML files. Briefly, all searches required a 10-ppm precursor mass tolerance, 0.02-Da fragment mass tolerance, strict tryptic cleavage, up to 2 missed cleavages, fixed modification of cysteine alkylation, variable modification of methionine oxidation, variable modification of phosphorylation on serine, threonine and tyrosine for the phosphopeptide-enriched samples and protein-level expectation value scores of 0.0001 or lower. Protein sequence libraries were built monthly from the most current UniProtKB distribution [21]. The mouse library from the January 2020 UniProt build was used. MGF files were searched using X!Tandem [31] and Comet [32]. XML output files were parsed using BiblioSpec [33], and nonredundant protein sets were determined using Proteome Cluster based on previously published rules [34]. MS1-based isotopic features were detected, and peptide peak areas were calculated using OpenMS [30]. Proteins were required to have 2 or more unique peptides across the analysed samples with E-value scores of 0.0001 or less. Fold change and differential expression were evaluated using MS-EmpiRe [35].

### Acetyl-CoA Quantifications

These were carried out as described [36]. Briefly, one hemisphere was homogenized on ice in a Dounce homogenizer (8 strokes per sample) in 2 ml of isolation buffer (10 mM Tris, pH 7.4, containing 166 mM sucrose and 1 mM EDTA) supplemented with a cocktail of protease inhibitors (Thermo Fisher Scientific #A32963). Brain homogenates were then subjected to centrifugation (1300 g, 4 °C, 5 min), and the supernatants obtained were placed in separate tubes. Pellets were resuspended in 1 ml of isolation buffer and spun down (1300 g, 4 °C, 5 min). Supernatants from the 1st and 2nd centrifugations were pooled, and ~ 0.1 ml (or 1 mg of total protein) from these combined supernatants was used for acetyl-CoA measurements by a kit (Sigma-Aldrich, MAK039) according to the manufacturer’s instructions. Of the remaining supernatants, 2 ml was subjected to centrifugation (21,000 g, 4 °C, 10 min), and the resulting pellets were resuspended in 1 ml of 15% Percoll. Suspensions were placed on the Percoll density gradient in a centrifuge tube prepared by layering 23% Percoll (3 ml) over 40% Percoll (3 ml). Enriched mitochondrial fractions (approximately 0.5 ml) were obtained after centrifugation (30,700 g, 4 °C, 5 min) as a band at the interface of 23% and 40% Percoll. This band was removed and mixed with 2 ml of isolation buffer containing 0.02% digitonin. Mitochondrial suspensions were spun down (16,700 g, 4 °C, 10 min), and the mitochondria obtained were washed by resuspension in 1.5 ml of isolation buffer and another centrifugation (6900 g, 4 °C, 10 min). The mitochondria were then resuspended in 0.3 ml of isolation buffer, homogenized and used (0.1 ml or 0.4 mg) for the determination of the acetyl-CoA concentration as described for brain homogenates.

### Metabolomic Profiling

One brain hemisphere (~ 200 mg, the weight of each hemisphere was recorded) was homogenized in 5 ml of the chloroform:methanol mixture (2:1, V/V). Homogenates were spun down at 4000 rpm and 4 °C for 10 min, and supernatants were removed into other tubes. Brain extraction was repeated with another 5 ml of the chloroform:methanol mixture, and the supernatants were combined and partially evaporated to 2 ml. Metabolites were further extracted by the addition of 1 ml of water to form a water–methanol layer, which was removed and evaporated to dryness. Dry residue was reconstituted in 400 µl of 80% methanol, and 100 µl was removed for the glucose assay as described below. The remaining 300 µl was spiked with internal standard (heptadecanoic acid, 0.1 mg/ml) and evaporated to dryness. Metabolites were converted to their methoximated derivatives by incubations with 100 µl of methoxylamine in pyridine (15 mg/ml) at 70 °C for 2 h. Samples were then evaporated to dryness, and 70 µl of trimethylsilyl trifluoroacetamide with 10% trimethylchlorosilane (Regisil, Regis Technologies) was added and heated at 70 °C for 30 min. Silylated metabolites were injected into an Agilent 5973 mass spectrometer equipped with an Agilent 6890 gas chromatograph. Relative metabolite concentrations were determined as a ratio of metabolite abundance to standard abundance. For glucose measurements, a 100 µl aliquot was spiked with internal standard ([U-^13^C]glucose (1 mg/ml), transferred to gas chromatography (GC)-MS vials and evaporated to dryness. Acetic anhydride (150 µl) in pyridine (2:1, V/V) was added to the vials to convert glucose to its pentaacetate derivative by reacting at 60 °C for 30 min. Solutions were evaporated to dryness and reconstituted in 80 µl of ethyl acetate. Samples were transferred to GC–MS inserts and crimped. Sample analyses were performed in duplicate by injecting 1 μl into the GC–MS instrument. The m/z values of 200 (M0) and 205 (standard) were monitored, and relative levels of glucose were determined using the internal standard. Metabolites were separated using an HP-5MS capillary column (60 m × 0.25 mm × 0.25 μm, Agilent Technologies, Santa Clara, CA) with a helium flow of 1.5 ml/min. Samples were analysed in selected ion monitoring mode using electron impact ionization; the ion dwell time was set to 10 ms. The following metabolites were determined: lactate (m/z 219), alanine (m/z 116), glycerol (m/z 218), glycine (m/z 248), succinate (m/z 247), fumarate (m/z 245), malate (m/z 233), serine (m/z 218), aspartate (m/z 232), α-ketoglutarate (m/z 247), glutamine (m/z 246), glycerol-3-phosphate (m/z 445), myristic acid (14:0, m/z 285), palmitic acid (16:0, m/z 313), stearic acid (18:0, m/z 241) and oleic acid (18:1, 339).

### Statistics

Data from all available brains were used. There were no exclusions of statistical outliers. The data represent the mean ± SD. A two-tailed, unpaired Student’s t-test was used for metabolomic profiling; the sample size is indicated in each figure or in the figure legend. Statistical significance was defined as *P ≤ 0.05, and **P ≤ 0.01 and ***P ≤ 0.001.

## Results

### Altered Protein Abundance in the Brains of EFV-Treated 5XFAD Mice From the 2TP

**Fig. 2:**
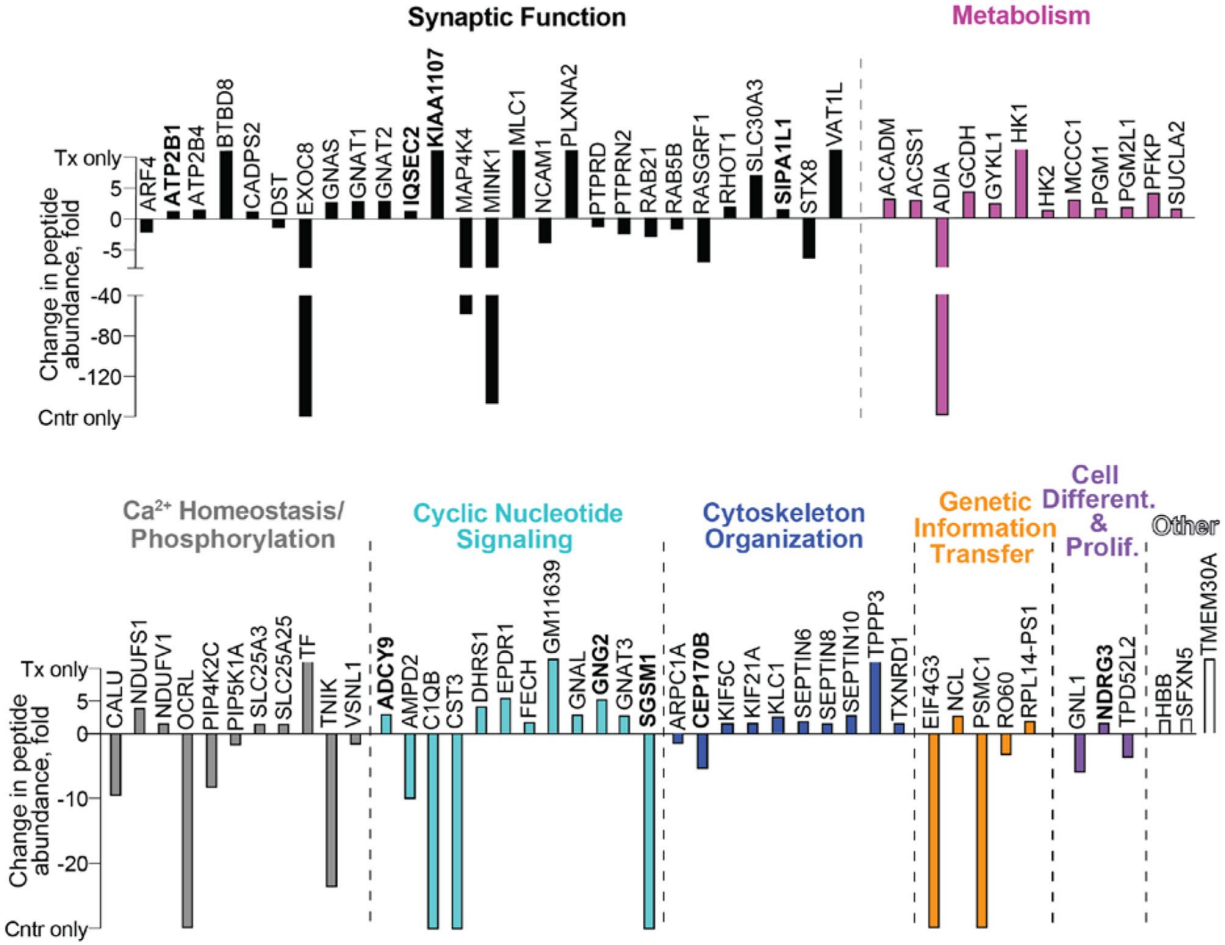
Differentially abundant proteins in EFV-treated (Tx) vs control (Cntr) 5XFAD mice from the second treatment paradigm. Synaptosomal fractions were used. Protein grouping is by process and shows each protein in only one group despite its involvement in multiple processes. Proteins in bold are those that also had altered phosphorylation

**Fig. 3:**
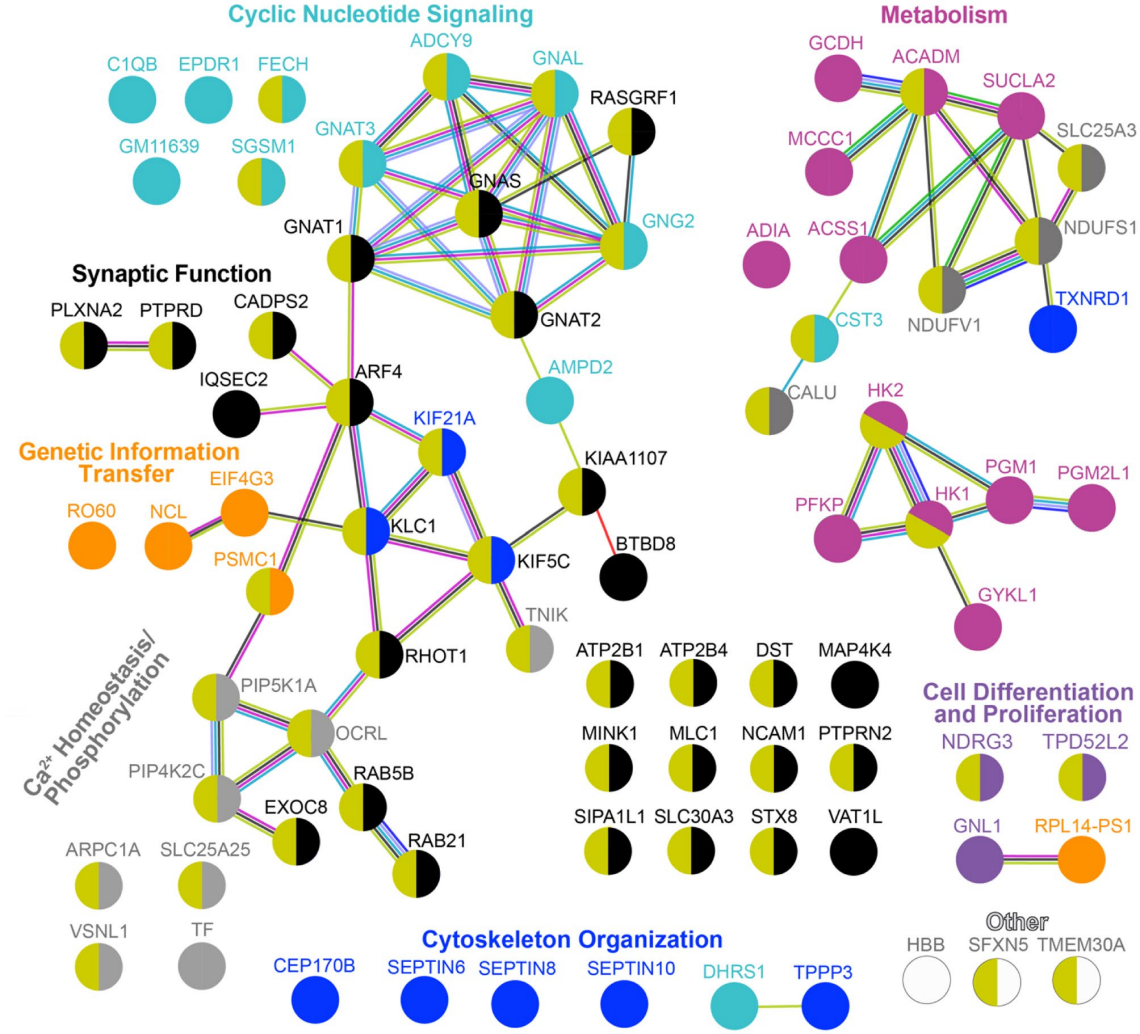
STRING analysis of the differentially abundant proteins in synaptosomal fractions from EFV-treated vs control 5XFAD mice on the second treatment paradigm. Nodes represent individual proteins and are coloured to indicate a functional group (the colour varies for each group) and interaction (direct or indirect) with the membrane (olive colour for all membrane-associated proteins). Association networks are shown as nodes linked by lines, which indicate known or experimentally determined protein–protein interactions (magenta), co-expression (black), co-mentioning in PubMed (lime); availability of experimental data (light purple); protein homology (light blue); predicted gene co-occurrence (blue); known interaction from curated databases (cyan); gene fusion (orange); and neighbourhood in genome (green)

**Fig. 4:**
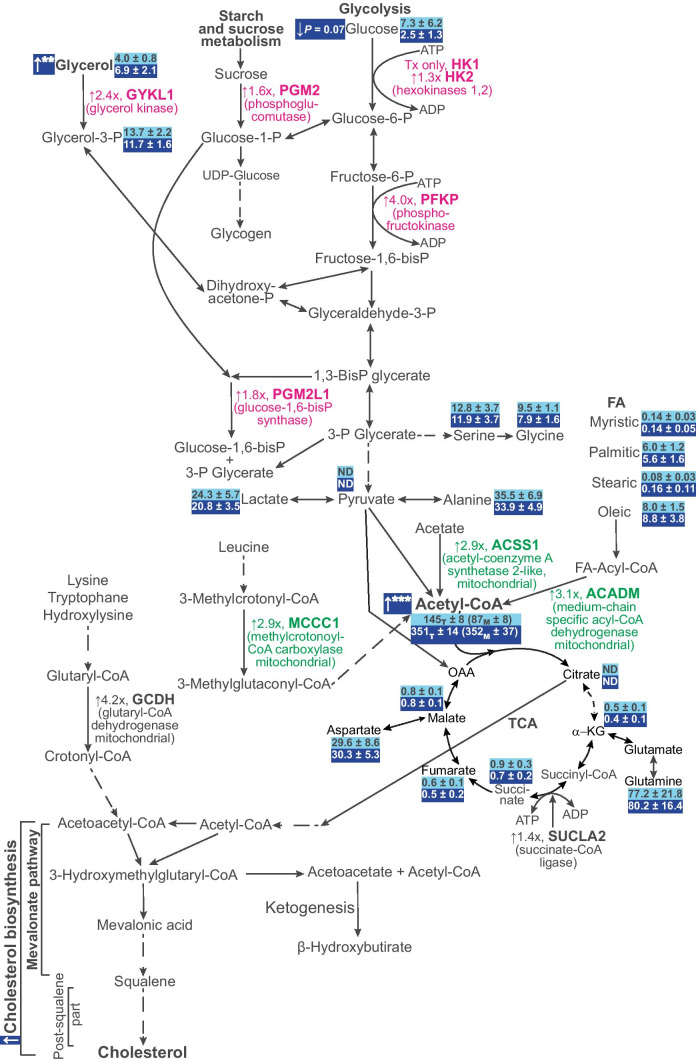
A summary of differentially abundant metabolic proteins, acetyl-CoA levels and metabolic profiling in the brains of EFV-treated vs control 5XFAD mice from the second treatment paradigm. Proteins involved in glycerol and sugar metabolism are bolded and indicated in magenta, and those involved in acetyl-CoA production are bolded and indicated in green. Upward and downward arrows indicate increases and decreases, respectively; x, fold change of EFV-treated vs control 5XFAD mice. The acetyl-CoA levels are shown in light blue boxes (control 5XFAD mice) and dark blue boxes (EFV-treated 5XFAD mice) as picomoles per milligram of total brain (T) or mitochondrial (m) protein (in parentheses). The metabolite levels are shown as micromolars per gram of brain tissue. Dashed arrows indicate multiple steps. FA, fatty acids; α-KG, α-ketoglutarate; OAA, oxaloacetate; P, phosphate, TCA, the tricarboxylic acid cycle

Synaptosomal fractions rather than brain homogenates were used because our previous studies of brain homogenates by the label-free approach were not successful, possibly because the brain is very rich in lipids, which interfere with protein extraction and subsequent processing. However, focusing on synaptosomal fractions from the whole brain also has limitations, as these fractions are comprised of different types of neurons across several brain regions. Accordingly, synaptosomal proteomics may not be sensitive enough to identify all of the affected proteins. Nevertheless, a total of 13,295 peptides from 4108 proteins were identified. Of them, 83 (including three serine/threonine protein kinases—MAP4K4, MINK and TNIK, and two receptor-type tyrosine-protein phosphatases—PTPRD and PTPRN2) were differentially abundant in EFV-treated vs control 5XFAD mice: 53 proteins had increased abundance and 30 proteins had decreased abundance (Fig. 2). Over one-third or 27 of differentially abundant proteins pertained to synaptic function, and the remaining 56 were involved in metabolism, Ca^2+^-homeostasis/phosphorylation and cyclic nucleotide signalling (12 proteins in each group) as well as cytoskeletal organization (9 proteins), genetic information transfer (5 proteins), cell differentiation and proliferation (3 proteins) and other processes (3 proteins). The differentially abundant proteins formed 5 major association networks according to STRING [37], three of which were interlinked and encompassed proteins important for synaptic function, cyclic nucleotide signalling, cytoskeletal organization and Ca^2+^-homeostasis/phosphorylation (Fig. 3). Notably, the other two association networks pertained mainly to metabolism and included energy metabolism and the production of acetyl-CoA, a substrate for the first step in the cholesterol biosynthesis pathway (Fig. 4). Remarkably, all of the differentially expressed metabolic proteins (except ADIA) had increased abundance in EFV-treated vs control 5XFAD mice, thus suggesting a change in the abundance of acetyl-CoA (Fig. 4).

### Altered Acetyl-CoA Levels in the Brains of Mice with Modulated Sterol Fluxes

**Fig. 5:**
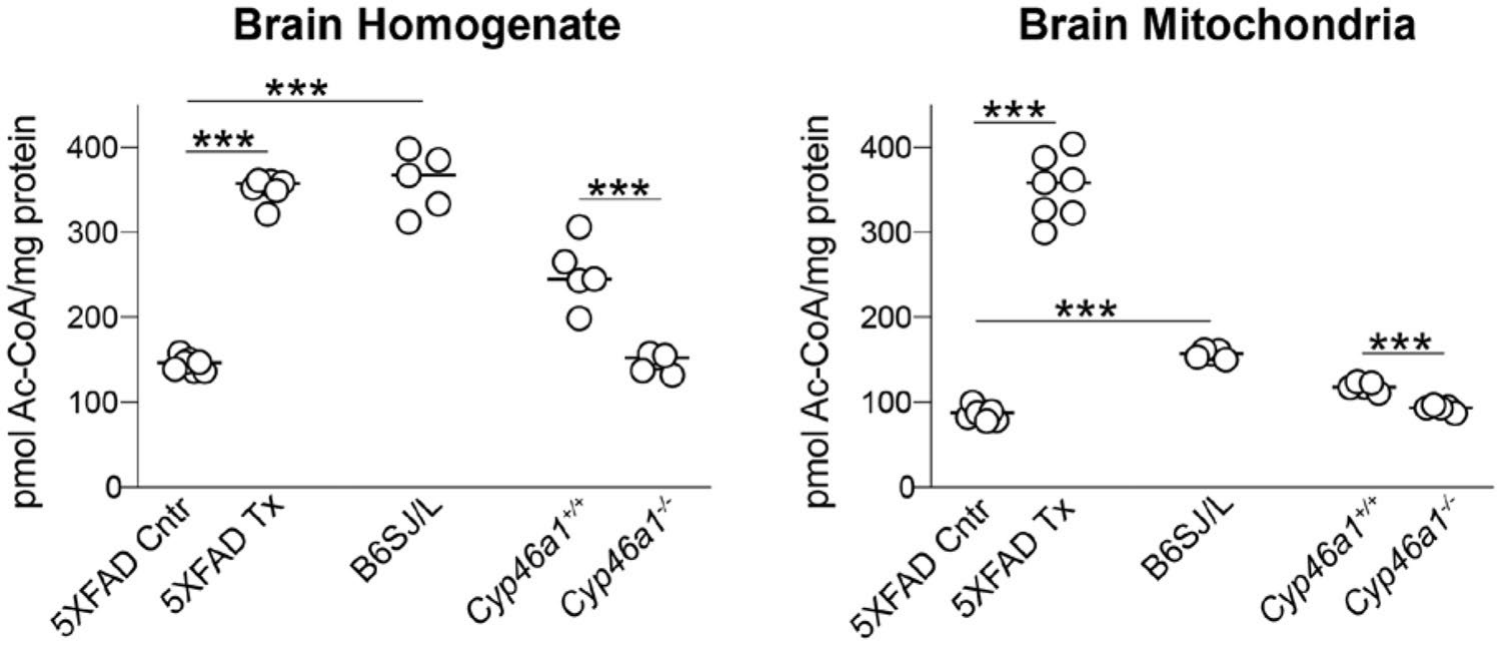
Effects of sterol flux on the levels of acetyl-CoA. EFV-treated (Tx) 5XFAD vs control (Cntr) 5XFAD mice had increased sterol flux, *Cyp46a1*^*−/−*^ vs *Cyp46a1*^+*/*+^ mice had decreased sterol flux, and B6SJL mice had a normal sterol flux. The results are the mean ± SD of measurements in individual animals: Cntr and Tx 5XFAD mice from the second treatment paradigm (n = 7); B6SJ/L mice (n = 5); *Cyp46a1*^*−/−*^ and *Cyp46a1*^+*/*+^ mice (n = 5). A two-tailed, unpaired Student’s t-test was used for statistical analyses. ***P ≤ 0.001

Acetyl-CoA is a membrane-impermeable molecule and, as such, forms separate mitochondrial, peroxisomal, nucleocytosolic and intrareticular pools [38]. We first assessed the total and mitochondrial acetyl-CoA pools in EFV-treated vs control 5XFAD mice from the 2TP. Both pools were increased 2.4-fold in the brain homogenates and 4.1-fold in the mitochondrial fraction (Fig. 5). Next, we measured the acetyl-CoA levels in the background B6SJL strain to gain insight into whether the observed increases in 5XFAD mice represent a positive or a negative EFV effect. The total acetyl-CoA content in B6SJL mice was similar to that in EFV-treated 5XFAD mice, and the mitochondrial content was 2.2-fold lower. This result suggested that EFV treatment had a normalizing effect on the total acetyl-CoA content in 5XFAD mice and increased the energetic state of the brain mitochondria. The latter is because acetyl-CoA contains an energy-rich thioester bond and is utilized in the mitochondria in different processes, including the tricarboxylic acid cycle (TCA), which yields NADH and FADH_2_, the substrates for ATP synthesis in oxidative phosphorylation [38]. Finally, *Cyp46a1*^*−/−*^ vs wild-type mice were assessed and found to have a decrease in both total and mitochondrial acetyl-CoA pools. This change was in the opposite direction to that of EFV-treated vs control 5XFAD mice, thus indicating that there is a link between acetyl-CoA levels and CYP46A1 activity or the rate of sterol flux.

### Altered Metabolomic Profile of the Brains of EFV-Treated 5XFAD Mice on the 2TP

Cellular levels of acetyl-CoA reflect a balance between different pathways of input (i.e., multiple catabolic reactions) and output (i.e., various anabolic reactions, including cholesterol biosynthesis) (Fig. 4) [38]. Previously, we showed that EFV treatment increased cholesterol biosynthesis in wild-type and 5XFAD mice [13]; specifically, it affected a pathway of acetyl-CoA output. Herein, we focused on the pathways of acetyl-CoA input and the three major catabolic circuitries that lead to the production of acetyl-CoA. We measured the levels of 19 metabolites: glycerol and glycerol-3-phosphate from glycerol metabolism; myristic, palmitic, stearic and oleic acids, the major substrates in fatty acid β-oxidation; glucose, pyruvate and lactate from glycolysis; serine, glycine, aspartate and glutamine from amino acid production; and citrate, α-ketoglutarate, succinate, fumarate, malate and oxaloacetate from the TCA (Fig. 4; Suppl. Figure 1). The levels of glucose were decreased almost threefold in EFV-treated vs control 5XFAD mice, consistent with an increased abundance of HK1, HK1 and PFKP, the latter being the rate-limiting enzyme in glycolysis [39], and an unaltered peptide abundance of GLUT1 and GLUT3, the two major glucose transporters in the brain [40]. However, this decrease was at the level of a trend (*P* = 0.07) because of significant data variability in the control group. In addition, there was a 1.7-fold increase in glycerol levels and no change in glycerol-3-phosphate levels, despite the increased abundance of GYKL1, which converts glycerol into glycerol-3-phosphate. Perhaps glycerol-3-phosphate utilization was increased as well. The levels of all other measured metabolites were unchanged, and those of pyruvate and citrate remained undetectable, although we previously detected pyruvate and citrate in the mouse retina, which, like the brain, is a neural tissue [41]. Thus, an increase in the total acetyl-CoA pool in EFV-treated vs control 5XFAD mice could be due to an increase in acetyl-CoA production as a result of increased glucose utilization, normally the principal source of acetyl-CoA and energy in the brain [42], and possibly glycerol.

### Differentially Phosphorylated Proteins in the Brains of 5XFAD Mice From the 2TP

This cohort of animals was different from that in the 1TP, which was analysed previously [7, 18]. In the 2TP, the duration of EFV administration was shorter (6 vs 8 months), and started at a mouse age of 3 months rather than 1 month age [20]. A total of 251 phosphopeptides from 211 proteins (brain homogenates) and 30 phosphopeptides from 24 proteins (synaptosomal fractions) had statistically significant differences in abundance in EFV-treated vs control 5XFAD mice from the 2TP (Suppl. Tables 1 and 2). Most of the affected proteins and peptides differed in the brain homogenates and synaptosomal fractions, while 7 proteins (AP3D1, GAD1, MAP1A, MAP1B, MAP2, NEFH and RIMS1) and 1 peptide (from GAD1) overlapped. Hence, we combined the two datasets into one, which was then used for subsequent analysis and comparisons. In this combined dataset, 152 and 128 phosphopeptides had increased and decreased abundance, respectively, in EFV-treated vs control mice, a reflection of increased and decreased phosphorylation of 124 and 93 proteins, respectively. Among 17 proteins, phosphopeptides with both increased and decreased abundance were present. Nine of the differentially phosphorylated proteins (ATP2B4, ADCY9, CEP170B GNG2, IQSEC2, KIAA1107, NDRG3, SGSM1 and SIPA1L1) also had altered abundance (Fig. 2). The two largest groups of differentially phosphorylated proteins pertained to synaptic function (~ 38% or 89 proteins) and cytoskeletal organization (8% or 18 proteins) (Table 1), and these proteins encompassed the aspects of synaptic function previously found in EFV-treated mice from the 1TP [18]. Thus, as with the 1TP, the 2TP altered protein phosphorylation in the brains of 5XFAD mice and affected similar processes.

**Table 1:** Differentially phosphorylated proteins (*P* ≤ 0.05) related to synaptic function and cytoskeletal organization in the brains of EFV-treated vs control 5XFAD mice from the 2TP. Each protein is placed in only one group despite involvement in multiple processes

Process
Synaptic function
Neurotransmitter synthesis, uptake, reception and downstream signalling
ADCY5, ADCY9, AGAP2, AKAP12, ALDH1A2, ARFGEF3, ELFN2, GABRA3, GAD1, GLUD1, GNG2, GRIA2, GRID2, GRIN2B, MATR3, PAM, PNPO, RGS7BP, SERINC1, SHISA7, SlC9A3R1, SLC18A2, TSPYL2
Synaptic vesicle exocytosis and trafficking
AP3D1, BSN, CADPS, CHGA, CHGB, DNAJC6, ERC1, FMN2, GIT1, ITSN1, KCNC1, KIAA1107, PCLO, PIP5K1C, RGS12, RIMS1, SLC4A8, STXBP5, STXBP5L, SYT1, VPS50
Axonal and neurite growth
ARFGEF1, BASP1, CCDC120, DCLK1, FGFR1, GPM6A, GPRIN1, PLCD1, PLPPR4, PLXNA1, PUM2, RTN3, SNX16, TRIM2, ZFR, VGF
Postsynaptic spine maintenance, formation and excitability
ARHGAP23, ATP2B4, BCR, DBN1, DGKB, DMXL2, EPB41L2, ERBIN, FBXO2, GPR158, IQSEC2, KCNK1, KNDC1, LIMK1, PALM, PEX5L, PHF6, PRICKLE2, PRKCE, RALBP1, SEPT7, SHANK3, SIPA1L1, SPARCL1, STIP1, TAX1BP1, TRIO, TYRO3, VPS4B
Cytoskeletal organization
4.1, ANK2, BRSK2, CEP170B, CTNND2, DPYSL2, GAP43, MACF1, MAP1A, MAP2, MAP1B, MAP7D1, MAP7D2, MAPT, NEFH, NEFM, PHLDB1, SRCIN

### Common Differentially Phosphorylated Proteins in the Brains of 5XFAD Mice From the 1TP and 2TP

**Fig. 6:**
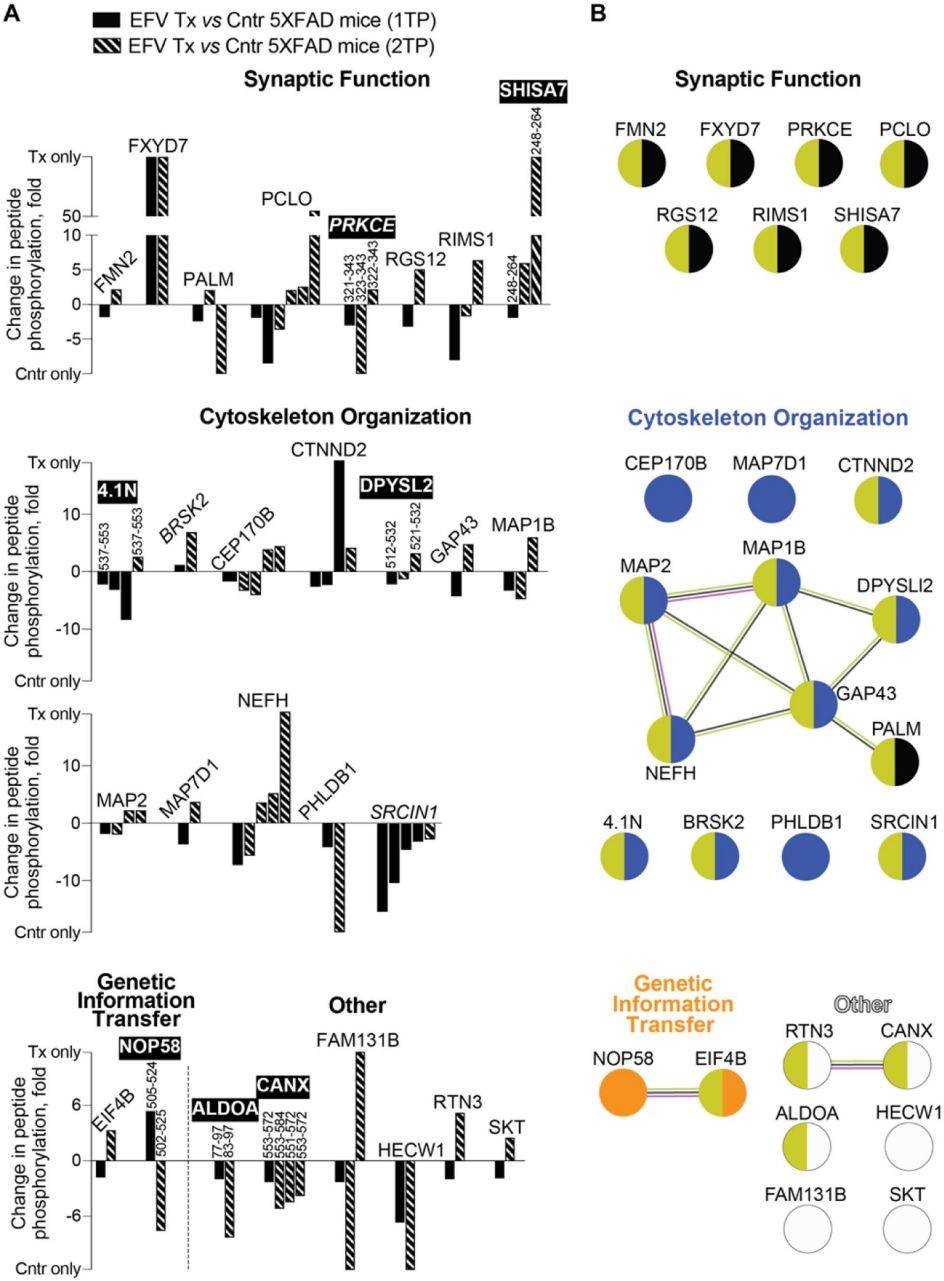
Differentially phosphorylated proteins common in EFV-treated (Tx) vs control (Cntr) 5XFAD mice from the first (1TP) and second (2TP) treatment paradigms. Protein grouping is by process and shows each protein in only one group despite its involvement in multiple processes. **A** Changes in peptide phosphorylation. Each peptide is shown as a separate bar and has amino acid sequence numbers above the bar if there is overlap in common differentially phosphorylated proteins. Proteins with overlapping phospho-sites are indicated in black boxes in bold white; protein kinases and a protein kinase inhibitor are indicated in italics. **B** STRING analysis of common differentially phosphorylated proteins. Nodes represent individual proteins, which are coloured according to function (the colour varies for each group) and interaction with the membrane (olive for all groups). Association networks are shown as nodes linked by lines, which indicate known or experimentally determined protein–protein interactions (magenta); co-expression (black); and co-mentioning in PubMed (lime)

If protein phosphorylation in the brain is indeed affected by the rate of sterol flux and is mediated in part by changes in membrane properties, then EFV-treated mice from the 1TP and 2TP should have common differentially phosphorylated proteins. A total of 28 such proteins, including two protein kinases (PKRCE and BRSK2) and one protein kinase inhibitor (SCRIN1), were found (Fig. 6A). These 28 common proteins pertained to at least three biological processes (synaptic function, cytoskeletal organization and genetic information transfer), and 10 of them were a part of the three association networks identified by STRING (Fig. 6B). The largest network encompassed proteins that were involved mainly in cytoskeletal organization, and the two small networks linked the proteins of importance for genetic information transfer (NOP58 and EIF4B) and the functions of the endoplasmic reticulum (ER) (RTN3 and CANX). The 28 common proteins had 82 peptides with altered phosphorylation (32 with increased abundance and 50 with decreased abundance), which overlapped in seven proteins (ALDOA, CANX, 4.1 N, DPYSL2, NOP58, PRKCE and SHISA7) (Fig. 6A).

To meet the requirements for the sterol flux effect, the overlapping differentially phosphorylated proteins in EFV-treated mice from the 1TP and 2TP should also have two other commonalities. First, the overlapping phosphoproteins and/or the enzymes that act on the affected phospho-sites should be associated directly or indirectly with the membrane. Second, the common phospho-sites should have changes in their phosphorylation in the same direction, namely, an increase or a decrease, compared to these sites in control mice. We found that 21 of the 28 overlapping differentially phosphorylated proteins were known to have either direct or indirect interactions with the plasma and/or ER membranes [37] (Fig. 6B), and among them, 2 proteins (ALDOA and CANX) had a decrease in phosphorylation of the overlapping peptides (Fig. 6A). In 5 proteins, the overlapping peptides had changes in phosphorylation in the opposite direction in mice from the 1TP and 2TP, yet these overlapping phosphopeptides had a different number of phosphorylated sites (e.g., 4.1 N, DPYSL2, and NOP58) and/or the phospho-sites were not always determined (e.g., PRKCE and SHISA7), making a direct comparison difficult (Suppl. Table 3). Thus, depending on how the 28 common phosphoproteins met our criteria for the sterol flux effect on protein phosphorylation, we suggest ALDOA and CANX as the strongest candidates for this effect, followed by 4.1, DPYSL2, NOP58, PRKCE and SHISA7 and finally the 21 remaining proteins.

### Common Differentially Phosphorylated Proteins in the Brains of Mice with Decreased and Increased Sterol Fluxes

**Fig. 7:**
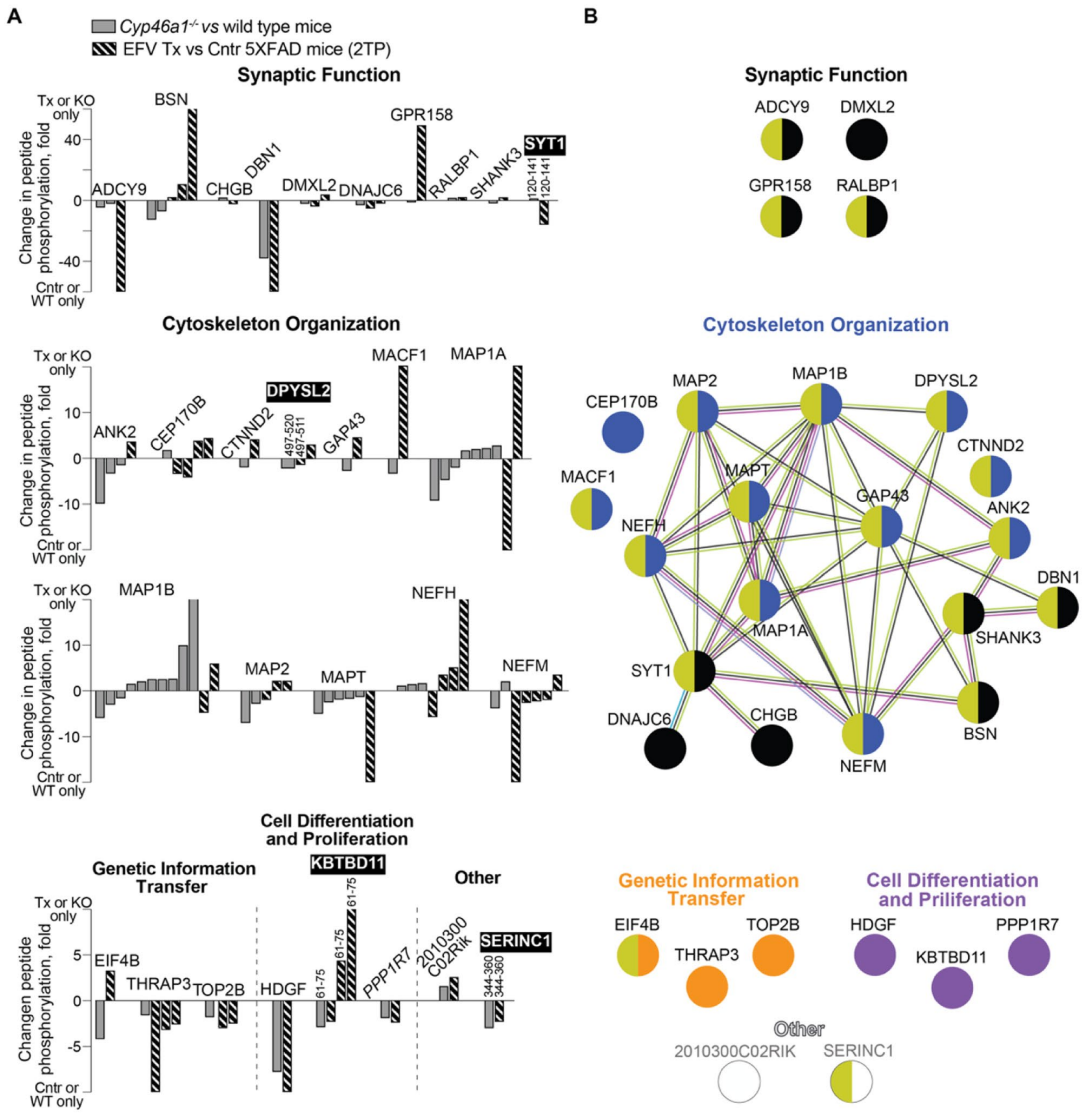
Differentially phosphorylated proteins common between *Cyp46a1*^*−/−*^ (KO) vs wild-type mice and EFV-treated (Tx) vs control (Cntr) 5XFAD mice on the second treatment paradigm (2TP). Protein grouping is by process and shows each protein in only one group despite its involvement in multiple processes. **A** Changes in peptide phosphorylation. Each peptide is shown as a separate bar and has amino acid sequence numbers above the bar if there is overlap among common differentially phosphorylated proteins; proteins with overlapping phospho-sites are indicated in black boxes and in white bold; the protein kinase is indicated in italics. **B** STRING analysis of common differentially phosphorylated proteins. Nodes represent individual proteins, which are coloured according to function (varies for each group) and interaction with the membrane (olive for all groups). Association networks are shown as nodes linked by lines, which indicate known or experimentally determined protein–protein interactions (magenta); co-expression (black), co-mentioning in PubMed (lime), and homology (light blue)

If protein phosphorylation in the brain is affected by the rate of sterol flux, then EFV-treated mice with decreased and increased sterol fluxes should also have common differentially phosphorylated proteins. Previously, we compared the differentially phosphorylated proteins in the brains of *Cyp46a1*^*−/−*^ vs wild-type and EFV-treated mice vs control 5XFAD mice from the 1TP [18], namely, animals in which the sterol flux rates were altered genetically and pharmacologically. Herein, we conducted a similar comparison of 5XFAD mice from the 2TP. The criteria for the proteins affected by the rate of sterol flux remained the same as that for mice with increased sterol flux from the 1TP and 2TP (see previous section), except we assumed that common differentially phosphorylated proteins do not necessarily need to have overlapping phospho-sites and that the phosphorylation change should be in the opposite direction. This was because we were comparing animals on different genetic backgrounds (C57BL/6 J;129S6/SvEv and B6SJL) and with very different genetic manipulations (gene ablation, *Cyp46a1*^*−/−*^ mice, and human mutant gene overexpression, 5XFAD mice), which could have different brain effects. A total of 30 common proteins were identified in the brains of *Cyp46a1*^*−/−*^ vs wild-type mice and EFV-treated vs control 5XFAD mice from the 2TP, including regulatory subunit 7 of protein phosphatase 1 (PPP1R7) (Fig. 7A). These 30 proteins pertained to synaptic function, cytoskeletal organization, genetic information transfer and cell differentiation and proliferation. Of these proteins, 12 formed one association network, which encompassed proteins important for synaptic function and cytoskeletal organization. Of the 30 common differentially phosphorylated proteins, 21 proteins interacted with the membrane (Fig. 7B), and four proteins (SYT1, DPYSL2, KBTBD11 and SERINC1) had overlapping peptides (Fig. 7A). The directionality of the phosphorylation change was in the opposite direction for SYT1 and KBTBD11, the same for SERINC1, and mixed for DPYSL2. Notably, only in SYT1 did the overlapping peptides have the same phosphorylated amino acid residue, whereas in DPYSL2, KBTBD11 and SERINC1, the combination of phospho-sites within the overlapping peptides was different (Suppl. Table 4). For comparison, 8 common differentially phosphorylated proteins were found in the brains of *Cyp46a1*^*−/−*^ vs wild-type mice and EFV-treated vs control 5XFAD mice from the 1TP, and two proteins (MAP6 and MAP1B) had overlapping phospho-sites [18].

**Fig. 8:**
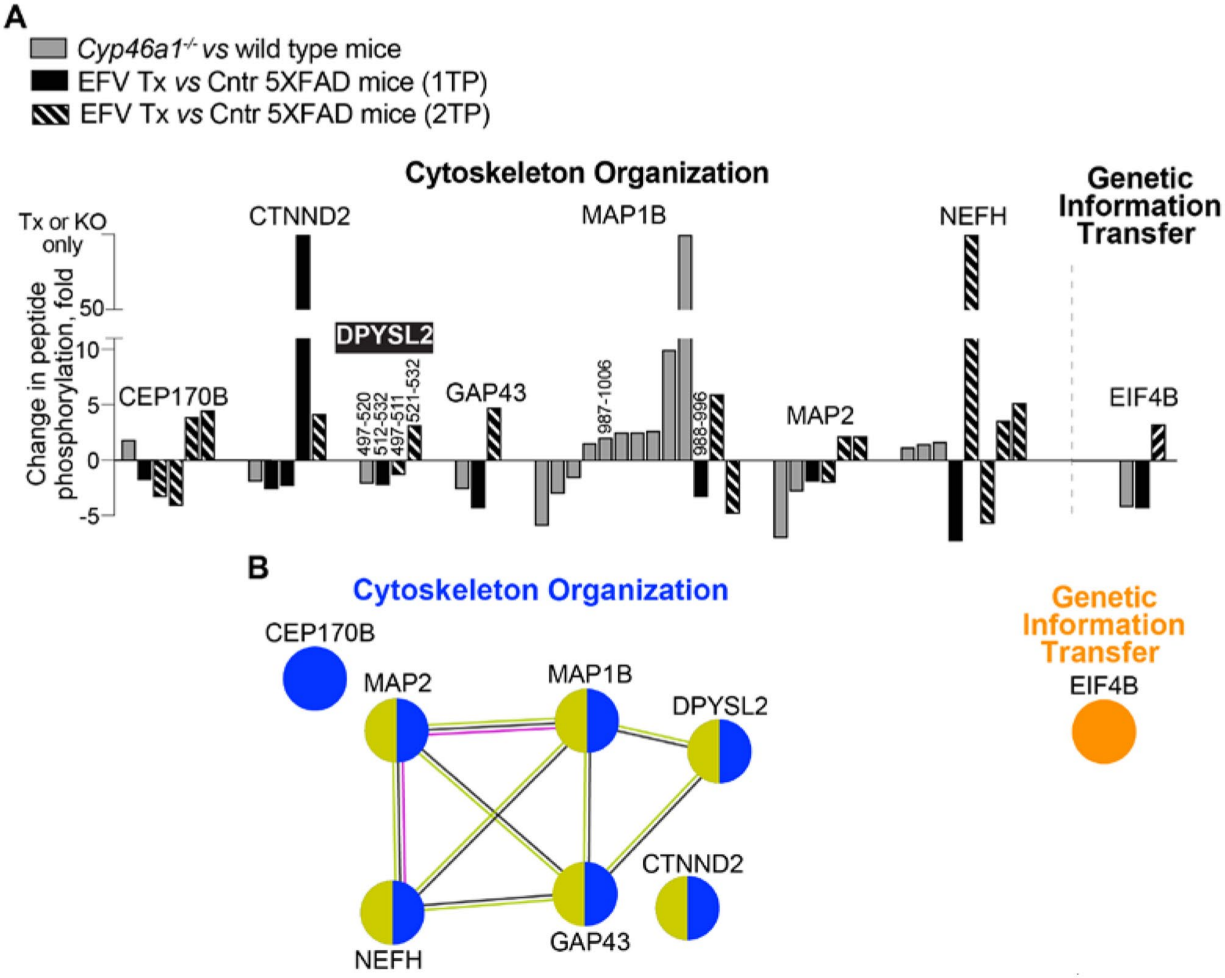
Differentially phosphorylated proteins common between *Cyp46a1*^*−/−*^ (KO) vs wild-type mice and EFV-treated (Tx) vs control (Cntr) 5XFAD mice from the first (1TP) and second (2TP) treatment paradigms. Protein grouping is by process and shows each protein in only one group despite its involvement in multiple processes. **A** Changes in peptide phosphorylation. Each peptide is shown as a separate bar and has amino acid sequence numbers above the bar if there is overlap among common differentially phosphorylated proteins; the protein with overlapping phospho-sites is indicated in black box and in white bold. **B** STRING analysis of common differentially phosphorylated proteins. Nodes represent individual proteins, which are coloured according to function (varies for each group) and interaction with the membrane (olive for all groups). Association networks are shown as nodes linked by lines, which indicate known or experimentally determined protein–protein interactions (magenta); co-expression (black); and co-mentioning in PubMed (lime). NEFM is a neurofilament component and one of the most phosphorylated brain proteins [93]. Phosphorylation stabilizes NEFM and promotes its assembly and interaction with microtubules [88]. CEP170B, a centrosomal protein, plays a role in microtubule organization by associating with spindle microtubules during mitosis, when it is also phosphorylated [94]. MAP2, the most abundant neural microtubule-associated protein, stabilizes, bundles, provides regular spacing between microtubules and helps direct microtubule motor transport [95]. At least 46 sites can be phosphorylated in MAP2, and in most cases, phosphorylation leads to MAP2 dissociation from microtubules [96]. MAP1B is a major growth-associated protein in neurons and glia. Phosphorylation of MAP1B, which has at least 35 phospho-sites [85, 97], influences microtubule stability, microfilaments and growth cone motility [85, 95]. DPYSL2, a multifunctional adaptor protein, is best known for its binding to tubulin heterodimers to promote microtubule assembly [98]. Perturbations in DPYSL2 function are found in numerous brain disorders [99]. DPYSL2 S522 phosphorylation facilitates subsequent phosphorylation of T509 and T514 [82] so that phosphorylated DPYSL2 loses affinity for tubulin heterodimers [82]. DPYSL2 is hyperphosphorylated at T509, T514, S518 and S522 in Alzheimer’s disease and aggregates in amyloid plaques and neurofibrillary tangles [98]. Coordinated DPYSL2 phosphorylation was uncoupled in EFV-treated 5XFAD mice from the 2TP (Suppl. Table 5) and was decreased in the T509-S518 region in other models of the altered sterol flux, probably a positive sterol flux effect. GAP43, a vertebrate-specific actin-interacting protein, is a marker of normal axonal growth and neural network formation [84, 100]. The S96 phosphorylation of GAP43 was decreased in *Cyp46a1*^*−/−*^ mice, an indication of impairments in these processes. CTNND2, a component of both adherens and synaptic junctions, can interact with actin and differentially regulate actin and microtubule remodelling [101]. Phosphorylation of CTNND2 T1078, which decreases protein stability [81], was increased in mice with increased sterol flux (Suppl. Table 5), which may be a regulatory effect, as CTNND2 has to be expressed at adequate amounts and correct locations to maintain normal brain functions [68, 81]. Only EIF4B does not pertain to the cytoskeleton, as this protein is involved in the initiation of protein synthesis by being a part of the ribosomal initiation complex [69, 70]. Phosphorylation increases EIF4 recruitment to the initiation complex, thus adjusting protein translation to neuronal needs and promoting adaptive changes in synaptic plasticity [83]

We next analysed whether there were common differentially phosphorylated proteins in the brains of *Cyp46a1*^*−/−*^ and EFV-treated 5XFAD mice from both treatment paradigms. Eight such proteins were identified, among which 7 pertained to cytoskeletal organization and one to genetic information transfer; five proteins formed an association network. Six common proteins are known to have direct or indirect interactions with the membranes (Fig. 8B), and one protein (DPYSL2) had overlapping phosphopeptides with altered phosphorylation in all three groups of mice (Fig. 8A). However, these peptides had a different combination of phospho-sites that were affected (Fig. 8; Suppl. Table 5). In addition, there was a peptide overlap in MAP1B, whose phosphorylation was decreased in EFV-treated 5XFAD mice from the 1TP and increased in *Cyp46a1*^*−/−*^ mice (Fig. 7). Thus, in all our comparisons of different phospho-proteomic datasets, there were common differentially phosphorylated proteins, including cytoskeleton-related proteins, further supporting our sterol flux hypothesis and providing unexpected mechanistic insight.

## Discussion

Herein, we found links between CYP46A1 activity (or the rate of brain sterol flux) and the levels of acetyl-CoA as well as phosphorylation of cytoskeletal proteins, our two major findings. The identification of the first link became possible due to unbiased proteomics and metabolomic profiling data. Indeed, EFV-treated 5XFAD mice, whose brain sterol flux was increased [13], had an ~ threefold increase in the abundance of ACADM, ACSS1 and MCCC1, the enzymes involved in acetyl-CoA biosynthesis, as well as increased expression of GYKL1, PGM2, PGM2L1, HK1, HK2 and PFKP, the enzymes that provide substrates for the synthesis of acetyl-CoA (Fig. 4). Consistent with the proteomics data, the total and mitochondrial acetyl-CoA levels were increased in EFV-treated 5XFAD mice and correlated with an increase or decrease in CYP46A1 activity, as shown by the measurements in *Cyp46a1*^*−/−*^ vs wild-type mice (Fig. 5). Additionally, in agreement with the proteomics data, the levels of glycerol and glucose were altered in EFV-treated 5XFAD mice, whereas the levels of the TCA intermediates remained unchanged (Fig. 4; Suppl. Figure 1). Either excess mitochondrial acetyl-CoA was utilized in this cycle or excess acetyl-CoA increased TCA turnover without affecting steady-state intermediate levels. The latter would be consistent with a 1.4-fold increase in the expression of SUCLA2, which catalyses the reversible conversion of succinyl-CoA to succinate (Fig. 4). Alternatively, and we favour this interpretation, a part of the acetyl-CoA excess entered the TCA by condensing with oxaloacetate to yield the citrate excess (Fig. 4), which was then quickly transported out of the mitochondria as normally occurs with glycolysis- or β-oxidation-derived mitochondrial acetyl-CoA, a major source of cytosolic acetyl-CoA following transportation [38]. In any case, once in the cytosol, mitochondrial citrate was probably canonically regenerated to acetyl-CoA and oxaloacetate, and acetyl-CoA was then used for different anabolic reactions [38]. Further studies are required to ascertain the acetyl-CoA fluxes in the mitochondria and cytosol in EFV-treated 5XFAD mice.

Two acetyl-CoA molecules are necessary to initiate the mevalonate pathway, an upstream (presqualene) part of cholesterol biosynthesis, and then a third acetyl-CoA molecule is required for the next step in this pathway, which is rate-limiting and irreversible (Fig. 4) [43]. Ultimately, 18 molecules of acetyl-CoA are used to produce one molecule of cholesterol, a product of 20 dedicated enzymatic reactions [44]. Cholesterol biosynthesis is believed to depend directly on acetyl-CoA levels [38, 42], and if so, it is an increased acetyl-CoA input that likely enables increased cholesterol biosynthesis in EFV-treated mice to compensate for increased cholesterol elimination. Cholesterol elimination and biosynthesis are known to be tightly coupled in the brain to maintain steady-state cholesterol levels [7, 13, 45]. However, the specific mechanism that maintains this coupling has not yet been investigated, perhaps because of the large amount of general knowledge of how cholesterol homeostasis can be maintained [46–48]. Herein, we identified acetyl-CoA as a molecule that couples cholesterol elimination and biosynthesis, a novel finding that enhances our understanding of cholesterol maintenance in the brain.

Cholesterol biosynthesis is an energy-consuming process that requires 18 ATP and 29 NADPH molecules per mole of cholesterol generated by metabolic circuitries that utilize acetyl-CoA [38, 44]. Hence, increased acetyl-CoA content in EFV-treated 5XFAD mice could be used not only to meet increased cellular demand for acetyl-CoA, a substrate for cholesterol production, but also for ATP and NADH, the energy sources for this anabolic process. Synaptic activity, plasticity and protein phosphorylation also require ATP, as its decreased production yields energy deficits and inhibits diverse reactions in neurons [49]. Consequently, increased acetyl-CoA production in EFV-treated 5XFAD mice could also contribute to their behavioural improvements and changes in protein phosphorylation. Another mechanism for behavioural improvements in EFV-treated 5XFAD mice could be acetyl-CoA involvement in the mevalonate pathway (Fig. 4), which is fundamental in memory function. Indeed, cognitive decline in *Cyp46a1*^*−/−*^ mice has been related to reduced amounts of a freshly synthesized nonsterol isoprenoid geranylgeraniol, which is produced in the mevalonate pathway and is required for long-term potentiation [50, 51]. Accordingly, the beneficial behavioural effects of EFV on 5XFAD mice could be due in part to the acetyl-CoA-dependent increase in metabolite flux through the mevalonate pathway.

Acetyl-CoA is a central molecule in many biological processes, including acetylation of choline and different proteins [49, 52]. The former generates acetylcholine in the presynaptic compartment, a neurotransmitter essential for memory and learning whose levels are decreased in Alzheimer’s disease [53]. Inhibition of acetylcholine breakdown by acetylcholinesterase with donepezil is currently a symptomatic treatment for Alzheimer’s disease [54]. It is conceivable that acetylcholine synthesis and, hence, cholinergic neurotransmission could be increased in EFV-treated 5XFAD and be another contributor to improvements in their behavioural tests in addition to the ATP and mevalonate pathways [7, 13]. Acetyl-CoA is a sole donor of acetyl groups for protein acetylation, including those of histones [52]. As such, acetyl-CoA controls gene expression via epigenetic regulation and alteration of the acetylation state of transcription factors [38, 55, 56]. Accordingly, the CYP46A1 activity effect on the acetyl-CoA levels could alter gene expression and thereby represent a general mechanism explaining changes in protein abundance found in EFV-treated 5XFAD mice characterized in the present work (Fig. 2). This effect would also explain changes in the brain transcriptome as a result of increased CYP46A1 activity reported previously [7, 10], and the data suggest epigenetic regulation of cholesterol homeostasis [48]. In addition, protein acetylation and acetyl-CoA control energy metabolism, mitosis, ER protein quality control and autophagy [38]. The latter was found to be affected by increased CYP46A1 expression in mouse models of Huntington’s disease and spinocerebellar ataxia [10, 12], consistent with increased acetyl-CoA production found in the present work. Further studies are required to determine whether protein acetylation is affected by CYP46A1 activity or brain sterol flux.

**Fig. 9:**
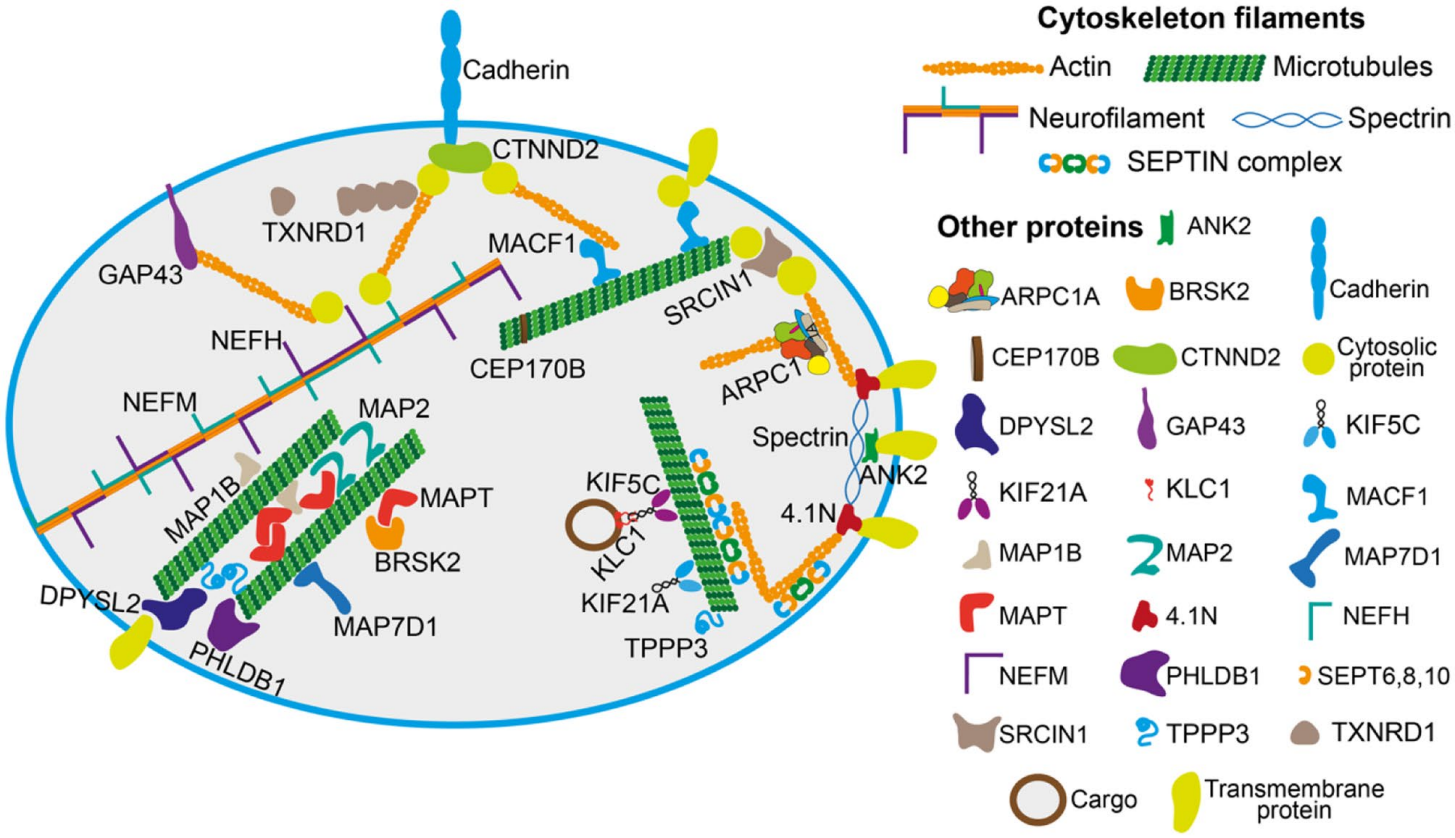
Cytoskeleton-related proteins whose phosphorylation or abundance was linked to changes in the rate of sterol flux. Of the differentially phosphorylated proteins, NEFM, MAP2, MAP1B, DPYSL2, GAP43, CTNND2 and CEP170B are already described in Fig. 8. In addition, NEFH, like NEFM, is a neurofilament component and one of the most phosphorylated brain proteins [93]. Phosphorylation stabilizes NEFM and promotes its assembly and interaction with microtubules. MAPT promotes microtubule assembly and stability and plays a role in axonal transport, synaptic structure and function and neuronal signalling [86]. Abnormal hyperphosphorylation and accumulation of MAPT are associated with Alzheimer’s disease and other neurodegenerative disorders known as tauopathies [102, 103]. SRCIN1 is an adaptor protein that indirectly interacts with microtubule plus ends to promote spine maturation. In addition, SRCIN1 interacts with the F-actin-binding protein and thus mediates cross talk between actin and microtubule dynamics, which are crucial for synaptic plasticity [104]. SRCIN1 is phosphorylated upon cell matrix adhesion and growth factor treatment [75]. Of the proteins with little data on their phosphorylation, 4.1 N is a membrane-cytoskeleton cross-linker and adaptor that bridges cytoplasmic spectrin-actin filament complexes and a wide variety of transmembrane proteins [105]. ANK2 is a membrane skeletal protein that contributes to mechanical support of plasma membranes by anchoring integral membrane protein complexes to the spectrin- and actin-based cytoskeleton [106]. BRSK2 is a brain-selective kinase required for neuronal polarization, likely because phosphorylation of its target microtubule-associated protein and subsequent changes in microtubule organization are critical for neuronal polarization [107]. MACF1 is an adaptor protein that can simultaneously bind to all three types of cytoskeletal fibres and connect with other adaptor and functional proteins [108]. MAP7D1 is a unique microtubule-associated protein that interacts with both microtubules and the motor protein kinesin-1 and is required for axon and branch growth [109]. PHLDB1 is an adaptor protein that anchors microtubule plus ends to the cell basal cortex [72]. Among the differentially abundant proteins, ARPC1A is the 1A subunit of the actin-related protein 2/3 complex that generates branched actin networks [110]. KIF5C, KIF21A and KLC1 are kinesins and motor proteins that mediate microtubule-directed transport [111, 112]. SEPTINS 6, 8 and 10 are GTPases from the same SEPTIN6 subgroup that form filaments and associate with actin and microtubule cytoskeletal networks [113]. When assembled, septins coordinate cell division and contribute to cell polarity maintenance and membrane remodelling [114]. TXNRD1 is a thioredoxin reductase and selenoprotein that displays multifaceted properties and functions beyond thioredoxin reduction [115]. A splice variant v3 of TXNRD1 forms cytoplasmic filaments and provokes the formation of cell membrane filopodia [116]. TPPP3 is an intrinsically unstructured protein that binds and polymerizes tubulin and stabilizes microtubules [117]

The present work revealed a link between sterol flux and the cytoskeleton, as cytoskeleton-related proteins had altered phosphorylation and abundance in EFV-treated vs control 5XFAD mice as well as in all our phospho-proteomics comparisons (Figs. 2, 6, 7 and 8). Collectively, the phosphoproteins that overlapped in our analyses encompassed two neurofilament components (NEFH and NEFM), three septin cytoskeletal components (SEP6, SEP8 and SEP10) and proteins that interact with these and other cytoskeletal structures (actin microfilaments, microtubules and the spectrin-rich membrane skeleton) (Fig. 9). Accordingly, almost every constituent of the cellular cytoskeleton could be affected by altered phosphorylation or abundance of phosphoproteins identified in the present work. Of particular importance was decreased phosphorylation of MAPT, a hallmark protein that is hyperphosphorylated in Alzheimer’s disease. The affected sites in mice with altered sterol flux included S648, S688, S692 and S696 (corresponding to S356, S396, S400 and S404, respectively, in the longest human tau isoform; Fig. 7; Suppl. Table 4), and they are all phosphorylated in advanced Alzheimer’s disease [57]. Decreased phosphorylation of these sites in *Cyp46a1*^*−/−*^ and EFV-treated 5XFAD mice is a positive effect of altered sterol flux. Neuronal size, shape, stability and communication depend on the cellular cytoskeleton [58]. Altered cytoskeletal phosphorylation in mice with altered sterol flux could explain in part why brain diseases such as neurodegenerative disorders, seizures, depression, and even cancer (glioblastoma) could benefit from CYP46A1 activity modulation [4–13].

The present work provided further evidence that altered sterol flux can affect protein kinases and phosphatases and thereby the brain phospho-proteome. We documented altered phosphorylation of two protein kinases (BRSK2 and PKRCE, Fig. 6), one negative regulator of protein phosphatase 1 (PPP1R7; Fig. 7) and one protein kinase inhibitor (SCRIN1; Fig. 6). Mice from the 2TP also had altered abundances of the protein kinases MAP4K4, MINK and TNIK and the protein phosphatases PTPRN and PTPRD (Fig. 6). In addition, we obtained evidence that the activity of several other protein kinases and phosphatases could be modulated, as some of their known substrates were differentially phosphorylated in mice with changes in CYP46A1 activity (Table 2). Notably, the putative effects on the activity of CDK5, GSK3 and PP1/PP2A were consistent with our previous work using protein kinase and/or protein phosphatase inhibitors [20]. Similarly, the effects on the activity of CK1, CK2, p38 MAPK, PKA and PKC were in strong agreement with our computational predictions based on the amino acid sequences of the differentially abundant phospho-sites in *Cyp46a1*^*−/−*^ mice and EFV-treated 5XFAD mice from the 1TP [18].

**Table 2:** Protein kinases and protein phosphatases that are known to act on some of the phospho-sites identified in the present work

Phospho-protein	Amino acid residue (enzyme)	Reference
CANX	S^563^ (ERK-1)	[80]
CTNND2	T^1078^ (GSK-3α/3β)	[81]
DPYSL2	S^522^ (CDK5); T^509^, T^514^ (GSK-3β)	[82]
EIF4B	S^504^ (CKs)	[83]
GAP43	S^96^ (JNK1)	[84]
MAP1B	S^1247^, S^1395^ (GSK-3β); S^1775^ (CDK5, CDC2); S^1778^ (PKA); S^1781^ (p38 MAPK)	[85]
MAPT	S^648^ (GSK-3, CK1, MARKS); S^688^ (GSK-3, CK1, CDK5, DYRK1A, AMPK); S^692^ (GSK-3, CK1, CDK5, DYRK1A); S^696^ (GSK-3, CK1, CDK5, DYRK1A, AMPK, JNK)	[57, 86]
NEFH	S^529,571,577, 757,763,769,809^ (all sites: MAPK1, MAPK2, PP1, PP2A); S^888^ (CDKs)	[87–89]
NEFM	S^502^ (CDKs)	
SYT1	T^128^ (CKII)	[64, 90, 91]

We continued to gain specific mechanistic insights. Previously, we found that synaptosomal fractions from EFV-treated 5XFAD mice had increased resistance to osmotic stress and membrane asymmetry, whereas those from *Cyp46a1*^*−/−*^ mice had changes in the opposite direction [20]. Herein, we documented altered abundance of MLC1 (Fig. 2, under synaptic function), which is a key player in response to osmotic challenge, and TMEM30A (Fig. 2, under other), an essential component of the P4-ATPase flippase complex, which maintains lipid asymmetry [59, 60]. Both MLC1 and TMEM30A were detected only in 5XFAD mice with increased sterol flux (Fig. 2) and hence could contribute in part to the changes in their membrane properties. Furthermore, the effects on exocytic glutamate release and postsynaptic density appearance were in the opposite direction in synaptosomal fractions from mice with increased and decreased sterol fluxes [20]. This could be due in part to the differential abundance of PIP4K2C, PIP5K1A (Fig. 2, under Ca^2+^-homeostasis) and EXOC8 (Fig. 2, under synaptic function) and altered phosphorylation of SYT1 (Fig. 6). Indeed, PIP4K2C and PIP5K1A sequentially phosphorylate phosphatidylinositol to produce phosphatidylinositol-4,5-bisphosphate, which is critical for synaptic vesicle exocytosis and endocytosis as well as many cellular events associated with the plasma membranes [61]. EXOC8 is a component of the exocyst, a protein complex involved in many cellular functions, including postsynaptic glutamatergic receptor trafficking and exocytosis [62]. SYT1 is a major synaptic vesicle membrane protein and Ca^2+^ sensor for synaptic neurotransmitter release [63]. The phosphorylation of SYT1 T128 [64] was inversely correlated with the rate of sterol flux (Fig. 7; Suppl. Table 4) and thus could affect neurotransmitter release. EFV treatment was also shown to inhibit glioblastoma growth and prolong the survival of mice with intracranial glioblastoma xenografts in previous studies [9]. Herein, we identified 6 cancer-related proteins with differential phosphorylation (4.1 N, CTNND2, EIF4B, PHLDB1, PRKCE and SRCIN1; Fig. 6) and 3 cancer-related proteins with altered abundance (GNL1, NDRG3 and TPD52L2; Fig. 2) [65–79]. It remains to be determined how the observed changes in these 9 proteins affect cancer.

In summary, we used the multi-omics approaches and metabolic profiling to characterize mice with changes in CYP46A1 activity and brain sterol flux. We found novel sterol flux effects on acetyl-CoA production and phosphorylation of the protein cytoskeleton, two processes that play key roles in cellular biology. Our findings enhance the biological significance of CYP46A1-mediated cholesterol 24-hydroxylation and provide an explanation for why CYP46A1 activity modulations are beneficial in a variety of mouse models of different diseases [4–13, 15]. We suggest that acetyl-CoA production could be another unifying factor, in addition to membrane properties, for the various sterol flux effects.

## Supplementary Information

Below is the link to the electronic supplementary material.

Supplementary file1 (PDF 543 KB)

Supplementary file2 (PDF 534 KB)

Supplementary file3 (PDF 543 KB)

Supplementary file4 (PDF 517 KB)

Supplementary file5 (PDF 525 KB)

Supplementary file6 (PDF 433 KB)

## Data Availability

Data will be made available on reasonable request. All data, materials and software applications comply with field standards.

## Abbreviations

1TP and 2TP: First and second treatment paradigms, respectively
acetyl-CoA: Acetyl coenzyme A
EFV: Efavirenz
ER: Endoplasmic reticulum
GC: Gas chromatography
LC: Liquid chromatography
MS: Mass spectrometry
TCA: Tricarboxylic acid cycle
UHPLC-MS/MS: Ultra-high-performance liquid chromatography tandem mass spectrometry
