# Supplementary material for: Brain Acetyl-CoA Production and Phosphorylation of Cytoskeletal Proteins Are Targets of CYP46A1 Activity Modulation and Altered Sterol Flux
Source: Neurotherapeutics. 2021 Jul 7;18(3):2040–60. doi: 10.1007/s13311-021-01079-6 (PMC8609074; doi:10.1007/s13311-021-01079-6)
Supplement: Supplementary file 6 — Supplementary file6 (PDF 433 KB) [file 13311_2021_1079_MOESM6_ESM.pdf]

**Supplemental Information**

# Brain acetyl-CoA production and phosphorylation of cytoskeletal proteins are targets of CYP46A1 activity modulation and altered sterol flux

Natalia Mast[1], Alexey M. Petrov[1,2], Erin Prendergast[3], Ilya Bederman[3] and Irina A. Pikuleva[1]

Department of [1]Ophthalmology and Visual Sciences and [3]Genetics and Genome Sciences, Case Western Reserve University, Cleveland, OH, USA.

[2]Current affiliations: Laboratory of Biophysics of Synaptic Processes, Kazan Institute of Biochemistry and Biophysics, Federal Research Center "Kazan Scientific Center of RAS", 2/31 Lobachevsky Street, box 30, 420111, Kazan, Russia; and Institute of Neuroscience, Kazan State Medial University, 49 Butlerova Street, 420012, Kazan, Russia

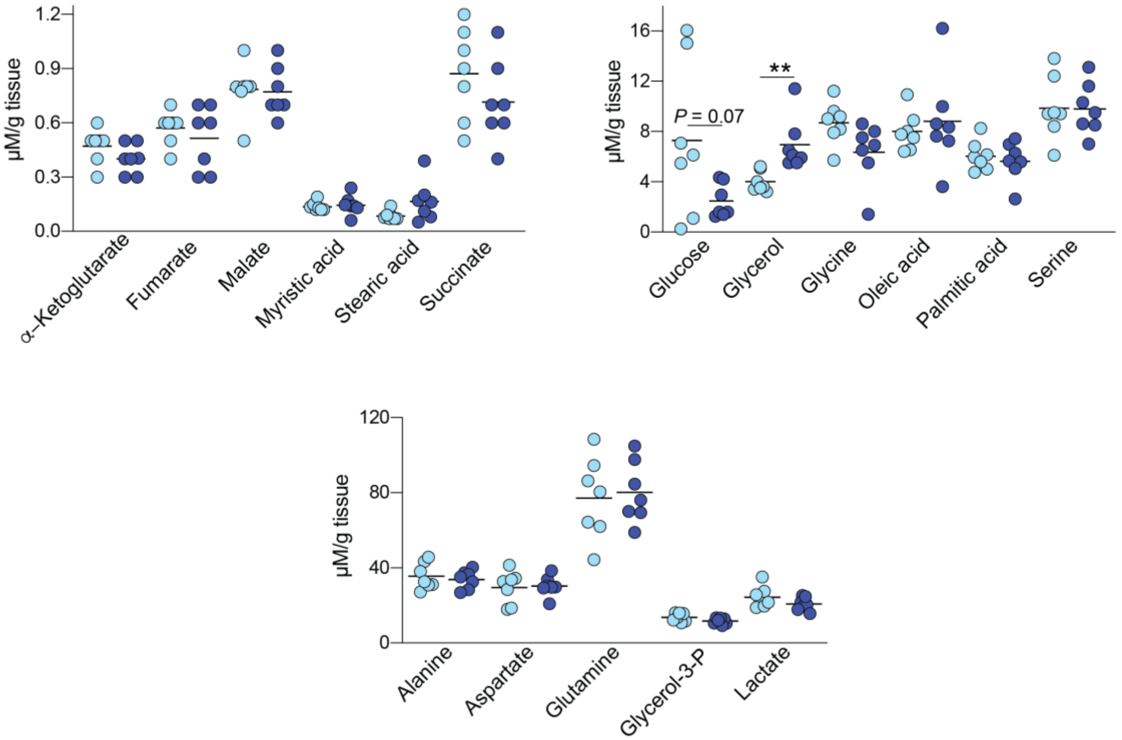


**Supplemental Fig. 1** The levels of different metabolites in the brain of EFV-treated (Tx) *vs* control (Cntr) 5XFAD mice from the 2TP. The results are the mean ± SD of measurements in individual animals: control (light blue dots) and EFV-treated (dark blue dots) 5XFAD mice (n=7). A two-tailed, unpaired Student's t-test was used for statistical analyses. **, $P \leq 0.01$.

**Supplemental Table 1** Phosphopeptides with statistically significant changes in abundance ($P \leq 0.05$) in the brain homogenates of EFV-treated (Tx) *vs* control (Cntr) 5XFAD mice from the second treatment paradigm. The identified phosphorylated amino acid residues (STY) are bolded, numbered and underlined. FC, fold change; Ace, acetylated amino acid residue; Mo, oxidized methionine.

| # | Protein | Peptide | FC, Tx/Cntr |
|---|---|---|---|
| **Proteins with a decreased phosphopeptide abundance** | | | |
| 1 | ADCY5 | SVSPPGYAAQTAASPAPR (1xPhospho [S/Y]) | -5.9 |
| 2 | ADCY9 | ASLG**S**$^{1295}$DDSTQAK | Cntr only |
| 3 | AHI1 | GKTPTADD**S**$^{26}$DDSREK | -2.2 |
| 4 | Akap12 | DKEADALPASTQEQDQAHGSSSPEPAGSPSEGEGVSTWESFK (2xPhospho [T/S]) | -2.0 |
| 5 | ALDH1A2 | IVG**S**$^{351}$PFDPTTEQGPQIDK (2xPhospho [S$^{351}$; T]) | -2.2 |
| 6 | API5 | TSEDTSSGSPPKK**S**$^{469}$PGGPK (2xPhospho [S$^{469}$; S]) | -2.6 |
| 7 | APPL2 | YVLLNDQADDTGG**S**$^{651}$PSENR | Cntr only |
| 8 | ATP2B4 | EAGHG**S**$^{1065}$DKEDISR | -2.1 |
| 9 | BNIP3 | NSTL**S**$^{88}$EEDYIER | Cntr only |
| 10 | CBX3 | RKSLSDSESDDSK (2xPhospho [S]) | -2.3 |
| 11 | CCDC120 | NSVASPTSPTR (1xPhospho [T/S]) | Cntr only |
| 12 | CD34 | LGEDPYYTENGGGQGYSSGPGA**S**$^{343}$PETQGK | Cntr only |
| 13 | CHGA | GELEH**S**$^{308}$QQEEDGEEAMVGTPQGLFPQGGK | -3.2 |
| 14 | CHGB | HIED**S**$^{190}$GEKPNTFSNK | -2.4 |
| 15 | CTNNA3 | SHTSIQTEGK (1xPhospho [T/S]) | -2.0 |
| 16 | CTR9 | RKG**S**$^{941}$G**S**$^{943}$EQEGEEEEGGER | -2.2 |
| 17 | DBN1 | **S**$^{385}$P**S**$^{387}$DSSTASTPIAEQIER | Cntr only |
| 18 | DNAJC6 | GASSPDMoEPSYGGGLFDMVK (1xPhospho [S])<br>CEEDHAALVNQE**S**$^{S478}$EQ**S**$^{481}$DDELLTLSSPHGNAEGDKPHGAK | -5.4<br>-2.1 |
| 19 | DPYSL2 | GLYDGPVCEV**S**$^{507}$V**T**$^{509}$PK | -1.3 |
| 20 | EIF3D | VYSLPDGTF**S**$^{528}$**S**$^{529}$EEDEEDEEEEEEEEEEET | -114.5 |
| 21 | ERBIN | STEDLSPQR (1xPhospho [S/T]) | -2.2 |
| 22 | FBXL3 | GGRD**S**$^{8}$DQD**S**$^{12}$AEEGTAEKPK | -2.7 |
| 23 | FBXO2 | NPCGEEDLEGW**S**$^{141}$DVEHGGDGWR | -2.7 |
| 24 | FGFR1 | SSTCSSGEDSVFSHEPLPEEPCLPR (2xPhospho [S]) | -2.7 |
| 25 | GATAD2B | LTPSPDIIVLSDNEASSPR (1xPhospho [S]) | -5.0 |
| 26 | GLUD1 | DDG**S**$^{128}$WEVIEGYR | -8.3 |
| 27 | GNG2 | EDPLL**T**$^{52}$PVPASENPFR | -2.1 |
| 28 | GPI | NRSNTPIKVDGK (1xPhospho [T/S]) | Cntr only |
| 29 | GRAMD1B | TESTYLAEIHRQ**S**$^{550}$PK | -4.1 |
| 30 | GRIA2 | EGYNV**Y**$^{876}$GIESVK | Cntr only |
| 31 | GRID2 | VP**S**$^{865}$KEDDKEIDLEHLHR | -3.3 |
| 32 | HDGF | GSAEGSSDEEGKLVIDEPAKEK (1xPhospho [S]) | Cntr only |
| 33 | HDGFL2 | AQEDGQD**S**$^{620}$EDGPR | -2.1 |
| 34 | HECW1 | GSTTEEEDGLEEESTLK (1xPhospho [T/S]) | Cntr only |
| 35 | HIST3H2BA | PEPSRSTPAPK (1xPhospho [T/S]) | -2.1 |
| 36 | HTATSF1 | AEEGGESEGDASEKDAK (1xPhospho [S]) | -2.1 |
| 37 | IRF2BP2 | **S**$^{250}$PADSLSSAAGASELSAEGAGK | Cntr only |
| 38 | KCNC1 | DAEEALDSFGGAPLDNSADDADADGPGDSGDGEDELEMTK (1xPhospho [S])<br>SVVNSPHHSTQSDTCPLAQEEILEINR (2xPhospho [T/S]) | -2.7<br>-2.4 |
| 39 | KCNK1 | QSEPFVASQSPPYEDGSADH (1xPhospho [S/Y]) | -3.2 |
| 40 | LARP7 | DLEFC**S**$^{334}$**T**$^{335}$EEEKETDR | -4.0 |
| 41 | LEO1 | KLN**S**$^{631}$DEEGESSGKR | -6.8 |

| | | | |
|---|---|---|---|
| 42 | LIMK1 | SCSIDTSPGTSSLASPASQR (1xPhospho [T/S]) | -2.5 |
| 43 | LUZP1 | VGNSGDAPEL**S**$^{982}$PR | -4.6 |
| 44 | MACROD2 | AKKPESSKDSSEDESGPEEK (2xPhospho [S])<br>KPESSKDSSEDESGPEEK (2xPhospho [S])<br>AKKPESSKDSSEDESGPEEK (3xPhospho [S]) | -3.6<br>-2.6<br>-2.4 |
| 45 | MAP1A | ELSSEPR**T** $^{T504}$PPAQK | Cntr only |
| 46 | MAPT | IG**S**$^{648}$LDNITHVPGGGNKK | Cntr only |
| 47 | MPHOSPH8 | GTVAVGDSEEDGEDVFEVER (1xPhospho [S/T]) | -2.3 |
| 48 | MTMR7 | S**S**$^{613}$DPDL**S**$^{618}$VNSDQESGVEDLSCR (3xPhospho [S$^{613}$; S$^{618}$; S])<br>SPSGGEHAPSEDSGKDR (1xPhospho [S]) | -3.3<br>-2.4 |
| 49 | NHEJ1 | DSGETQASSSTSPR (1xPhospho [T/S]) | Cntr only |
| 50 | NOP58 | HIKEEPL**S**$^{509}$EEEPCTSTAVPSPEKK | -7.6 |
| 51 | NUCKS1 | KDDSHSAEDSEDEKDDHK (1xPhospho [S])<br>DDSHSAED**S**$^{79}$EDEKDDHKNVR (2xPhospho [S$^{79}$; S])<br>DD**S**$^{73}$H**S**$^{75}$AED**S**$^{79}$EDEKDDHKNVR | -101.3<br>-3.6<br>-2.9 |
| 52 | OTUD4 | LQRPKEE**S**$^{1016}$**S**$^{1017}$EDENEVSNILR | -4.4 |
| 53 | PAM | VSTEGSDQEKDEDDG**S**$^{962}$ESEEEYSAPLPTPAPSS (3xPhospho [S$^{962}$; S]) | Cntr only |
| 54 | PAXBP1 | EDENDA**S**$^{264}$DDEDDDEKRR | -3.5 |
| 55 | PHF6 | TAHNSEADLEESFNEHELEPSSPK (1xPhospho [S]) | Cntr only |
| 56 | PHLDB1 | **S**$^{520}$P**S**$^{522}$PTLGESLAPR | Cntr only |
| 57 | PLCD1 | LGGLLPAGGENGPEATDVSDEDEAAEMEDEAVR (1xPhospho [S/T]) | Cntr only |
| 58 | PNPO | GLATGDSPLGPMTHHGEEDWVYER (1xPhospho [T/S]) | Cntr only |
| 59 | PPP1R7 | RVESEESGDEEGKK (1xPhospho [S]) | -2.4 |
| 60 | PRPF40A | HKSD**S**$^{881}$PE**S**$^{884}$DTEREK | -3.1 |
| 61 | RETREG2 | SAPPAGDEPLAETESESEAELAGFSPVVDVK(1xPhospho [T/S]) | -3.4 |
| 62 | RPS6KC1 | VCLQQPSASPQGGSSFESR (1xPhospho [S]) | Cntr only |
| 63 | RRP8 | ALEAA**S**$^{62}$L**S**$^{64}$QQTPSLPGSDSEEEEEVGR | Cntr only |
| 64 | SAMHD1 | TPPSTPPATANLSADDDFQNTDLR (1xPhospho [T/S]) | -2.8 |
| 65 | SERINC1 | LTLTSDESTLIEDGNGR (2xPhospho [S/T]) | -2.3 |
| 66 | SLC16A3 | F**S**$^{357}$**S**$^{358}$AIGLVLLLEAVAVLIGPP**S**$^{377}$GGK | Cntr only |
| 67 | SLC4A8 | DGQTV**S**$^{259}$PQSATNLEVK (2xPhospho [S$^{259}$; T/S]) | Cntr only |
| 68 | SMAP | SASPDDDLGSSNWEAADLGNEER (2xPhospho [S]) | -2.6 |
| 69 | SPARCL1 | HQSEQGNQGQE**S**$^{270}$D**S**$^{272}$EAEGEDK | Cntr only |
| 70 | STIP1 | HD**S**$^{481}$PEDVKR | -2.0 |
| 71 | SYT1 | DDDAETGL**T**$^{128}$DGEEKEEPKEEEK | -16.0 |
| 72 | TAX1BP1 | VP**S**$^{693}$WEDNVVCSQPAR | Cntr only |
| 73 | TCF20 | GSQEDDPAASQRPPSNSGVK (1xPhospho [S]) | Cntr only |
| 74 | THRAP3 | HGLTHEELK**S**$^{695}$PR<br>SSSKDSRPSQAAGDNQGDEAK (1xPhospho [S])<br>SY**S**$^{55}$PAHNR (2xPhospho [S$^{55}$; S/Y]) | Cntr only<br>-3.2<br>-2.6 |
| 75 | TMPO | SSTPLPTVSSSAENTR (2xPhospho [S/T]) | -13.0 |
| 76 | TOP2B | FDSNEEDTASVFAPSFGLK (1xPhospho [T/S])<br>SEDDSAKFDSNEEDTASVFAPSFGLK (1xPhospho [S/T]) | -3.0<br>-2.6 |
| 77 | TSPYL2 | SEVNSEDSDIQEVLPVPK (2xPhospho [S]) | -3.1 |
| 78 | VPS13C | EVSTPQDVHTTQGVPAAR (1xPhospho [T/S]) | -4.9 |
| 79 | VPS4B | GND**S**$^{102}$DGEAESDDPEKKK | -2.6 |
| 80 | ZC3H13 | LRSPSNDSAHR (1xPhospho [S]) | -2.4 |
| **Proteins with an increased phosphopeptide abundance** | | | |
| 81 | 1 SV | AD**S**$^{15}$E**S**$^{17}$EEDEQESEEVR | 4.3 |
| 82 | 4.1N | RLPSSPASPSPKG**T**$^{550}$PEK (3xPhospho [T$^{550}$; S]) | 2.1 |
| 83 | ADD3 | TEEVL**S**$^{673}$PDGSPSKSPSK (3xPhospho [S$^{673}$; S]) | 2.6 |
| 84 | AGAP2 | L**S**$^{211}$WPESEGKPR | 2.0 |
| 85 | ANK2 | EIASPSSPVK (2xPhospho [S]) | 3.7 |

| | | | |
|---|---|---|---|
| 86 | ANKRD63 | GSNSDSPPGHPAPAP**S**$^{203}$PER (2xPhospho [S$^{203}$; S]) | 2.6 |
| 87 | AP3D1 | RHSSLPTE**S**$^{760}$DEDIAPAQR (3xPhospho [S$^{760}$; S/T]) | 2.7 |
| 88 | APLNR | LSSYLIFVNMoYASVFCLTGL**S**$^{122}$FDR (3xPhospho [S$^{122}$; S/Y]) | 342.0 |
| 89 | ARFGEF1 | LSVSSNDTQESGNSSGPSPGAK (1xPhospho [T/S]) | Tx only |
| 90 | ARFGEF3 | H**S**$^{2074}$FSAGPELLR | 3.4 |
| 91 | ARHGAP23 | SKSCDDGLNTFR (1xPhospho [S]) | 8.3 |
| 92 | ARHGAP25 | TDSFSNTASSPDATSPTGPLPSDQHQEDSGK (2xPhospho [T/S]) | 9.7 |
| 93 | ARHGAP26 | SSDSKPPSCSK (1xPhospho [S]) | Tx only |
| 94 | ARPP21 | AWSSTDSDSSNR (1xPhospho [S/T]) | Tx only |
| 95 | ATP1A1 | DKYEPAAV**S**$^{16}$EHGDKK | 8.8 |
| 96 | ATP1A3 | KDDKSSPK (1xPhospho [S]) | 9.3 |
| 97 | BCR | SQ**S**$^{303}$TSEQEKR | Tx only |
| 98 | BRSK2 | G**S**$^{440}$PLP**T**$^{444}$PK | 7.0 |
| 99 | BSN | SQGSFEYQDTQDHDYGGR (1xPhospho [S])<br>SHGPLLPTIEDSSEEEELREEEELLREQEK (1xPhospho [S]])<br>QT**S**$^{3158}$LADLEQK | 2.2<br>10.8<br>Tx only |
| 100 | CADPS | LTVEEATASVSEGGGLQGISMKDSDEEDEEDD (1xPhospho [S]) | Tx only |
| 101 | CCDC92 | SATGQH**S**$^{261}$PAR | 336.8 |
| 102 | CDKN2AIP | SAAQQENSSPSR (1xPhospho [S]) | Tx only |
| 103 | CEND1 | ADPVLLNNHSNLKPAPTVPAAPSSPDATSEPKGPGDGAEEDESNTGGR (1xPhospho [S]) | Tx only |
| 104 | CHD2 | LKDEHGLEPA**S**$^{1365}$PR | Tx only |
| 105 | CIC | KNSTDLDSAPEDPTSPK (2xPhospho [S/T]) | 2.1 |
| 106 | CTNND2 | **T**$^{1078}$PSI**S**$^{1082}$PVR | 4.2 |
| 107 | DCLK1 | SGKSPSP**S**$^{334}$PT**S**$^{337}$PGSLRK | 3.0 |
| 108 | DDX21 | EIITEEP**S**$^{118}$EEEADMPKPK | 5.4 |
| 109 | DGKB | KG**S**$^{689}$DKRPTLTDAK | 14.8 |
| 110 | DNAL1 | LSL**S**$^{56}$TNCIEK | 5.7 |
| 111 | EIF4B | ARPTTDSFDDYPPR (1xPhospho [S/T]) | 3.3 |
| 112 | EIF4G1 | **S**$^{1187}$F**S**$^{1189}$KEVEER | 2.2 |
| 113 | ELFN2 | HYYSGYSSSPEYSSESTHK (2xPhospho [Y/S]) | 2.2 |
| 114 | ENAH | N**S**$^{351}$RPSSPVNTPSSQPPAAK (2xPhospho [S$^{351}$; S]) | Tx only |
| 115 | EPB41L2 | RD**S**$^{580}$KSPTK | Tx only |
| 116 | ERC1 | TNSTGGSSGNSVGGGSGK (1xPhospho [S/T]) | 6.4 |
| 117 | EVI5 | SD**S**$^{689}$NQYIR | 2.3 |
| 118 | FAM110B | SK**S**$^{257}$DLSDR | 4.00 |
| 119 | FAM171B | ILYLEDLDLSSSESGTTVCSPEDPALR (1xPhospho [S/T]) | 4.0 |
| 120 | FMN2 | **S**$^{720}$IQT**S**$^{724}$PTEEGR | 2.2 |
| 121 | FNBP1L | RHSSDINHLVTQGRESPEGSYTDDANQEVR (2xPhospho [S/T/Y]) | Tx only |
| 122 | FXYD7 | ADSRSESPTCK (2xPhospho [S]) | Tx only |
| 123 | GABRA3 | SAAAPSASSTPTAIASPK (2xPhospho [S/T]) | 2.5 |
| 124 | GAD1 | TN**S**$^{55}$LEEK | 3.0 |
| 125 | GAP43 | AGSAETESAAKATTDNSPSSK (1xPhospho [S]) | 4.8 |
| 126 | GIT1 | SQSELDDQHDYDSVASDEDTDQEPLPSAGATR (2xPhospho [S/T/Y]) | 3.1 |
| 127 | GOLPH3 | AAGGGGG**S**$^{35}$GEDEAQSR | 2.8 |
| 128 | GPR158 | RG**S**$^{239}$NQGPR | 49.5 |
| 129 | GRIN2B | RE**S**$^{929}$**S**$^{930}$VYDISEHR | 2.2 |
| 130 | IQSEC2 | ESQYQNLR (1xPhospho [Y89]) | 3.2 |
| 131 | KIAA1107 | **S**$^{714}$EDYDAGGSQDDEGSHDR (2xPhospho [S$^{714}$; S])<br>SSSDTSTPEELK (1xPhospho [S]) | 9.9<br>Tx only |
| 132 | KNDC1 | VVNGPASPSESTSEEPGSQPEHSPSR (1xPhospho [T/S])<br>AAASPSSPR (1xPhospho [S]) | 4.8<br>Tx only |
| 133 | LBH | LP**S**$^{63}$IVVEPTEGEVESGELR | Tx only |
| 134 | LDB2 | KNSTSSTSNSSAGNTTNSAGSK (1xPhospho [T/S]) | 4.8 |

| | | | |
|---|---|---|---|
| 135 | MACF1 | QG$\underline{\mathbf{S}}^{3889}$FSEDVISHK | Tx only |
| 136 | MAP1A | $\underline{\mathbf{S}}^{1762}$PFEII$\underline{\mathbf{S}}^{1768}$PPA$\underline{\mathbf{S}}^{1772}$PPEMoTGQR | Tx only |
| 137 | MAP1B | HMDPPPAPMQDR$\underline{\mathbf{S}}^{2205}$P$\underline{\mathbf{S}}^{2207}$PR | 6.0 |
| 138 | MAP4K3 | $\underline{\mathbf{S}}^{398}$VEEELHQR | 2.6 |
| 139 | MAP7D1 | AAEEKEPAAPA$\underline{\mathbf{S}}^{544}$PAPSPVPSPTPAQPQK (2xPhospho [$S^{544}$; S/T]) | 3.6 |
| 140 | MAP7D2 | VATSAASGGHG$\underline{\mathbf{S}}^{315}$PLR | 6.6 |
| 141 | MB21D2 | RGSTTSIPSPQSDGGDPNQPDDR (2xPhospho [S/T]) | 3.7 |
| 142 | MFF | ND$\underline{\mathbf{S}}^{146}$IV$\underline{\mathbf{T}}^{149}$P$\underline{\mathbf{S}}^{151}$PPQAR | 2.2 |
| 143 | MON1A | SYEDLTELEDR (1xPhospho [S/Y]) | 2.7 |
| 144 | MTFR1L | TTCSSSEEDDCISLSK (3xPhospho [S/T]) | 4.1 |
| 145 | MTMR12 | LIN$\underline{\mathbf{S}}^{601}$SDDLQDSSR | Tx only |
| 146 | NCOA7 | YY$\underline{\mathbf{S}}^{92}$VDDNQNK | 3.0 |
| 147 | NDRG3 | THSTSSSIGSGESPFSR (1xPhospho [T/S]) | 2.6 |
| 148 | NEDD4 | QI$\underline{\mathbf{S}}^{309}$EDVDGPDNR | 5.3 |
| 149 | NEDD4L | DTLSNPQ$\underline{\mathbf{S}}^{508}$PQPSPYNSPKPQHK (3xPhospho [$S^{508}$; S/Y/T]) | 2.5 |
| 150 | NEFH | $\underline{\mathbf{S}}^{571}$PAEAK$\underline{\mathbf{S}}^{577}$PAEPK | Tx only |
| 151 | NHSL1 | NH$\underline{\mathbf{S}}^{1373}$P$\underline{\mathbf{S}}^{1375}$PPVTPTSAAPNLASPK | 4.9 |
| 152 | NYAP2 | SQSLH$\underline{\mathbf{S}}^{123}$VGGTEDDSSCGSR | Tx only |
| 153 | OGFR | HEAK$\underline{\mathbf{S}}^{359}$P$\underline{\mathbf{S}}^{361}$PK | 248.7 |
| 154 | OSBPL10 | ATSAGS$\underline{\mathbf{S}}^{32}$PSCSLAGR | 2.8 |
| 155 | PACS2 | SHREPP$\underline{\mathbf{S}}^{319}$PADVPEK | 5.4 |
| 156 | PALM | TSTPVRSPGGSTMoMK (2xPhospho [T/S])<br>TSTPVRSPGGSTMMK (2xPhospho [T/S]) | 2.0<br>2.1 |
| 157 | PFDN2 | LMoGEDEKPAAKEN$\underline{\mathbf{S}}^{139}$EGAGAKA$\underline{\mathbf{S}}^{147}\underline{\mathbf{S}}^{148}$AGVLVS | Tx only |
| 158 | PIP5K1C | SSSLKSSPSK (1xPhospho [S]) | 3.4 |
| 159 | PITPNM1 | SSTEARPGTSTAGTPDGPEAPPGPDASPDASFGK (3xPhospho [S/T]) | Tx only |
| 160 | PITPNM2 | WN$\underline{\mathbf{S}}^{365}$NDLMDK | 2.0 |
| 161 | PLPPR4 | TVHTSPGGGLGAR (1xPhospho [S/T])<br>DSESCESLKDSFG$\underline{\mathbf{S}}^{685}$GDR | 2.0<br>2.6 |
| 162 | PLXNA1 | TAS$\underline{\mathbf{S}}^{1630}$PDSLR | 2.2 |
| 163 | PPM1H | GGVGAPGSPSTPPTR (1xPhospho [T/S]) | 2.7 |
| 164 | PRICKLE2 | $\underline{\mathbf{S}}^{732}$FQESLGQGS | 2.0 |
| 165 | PRPF4B | VEQE$\underline{\mathbf{S}}^{518}\underline{\mathbf{S}}^{519}\underline{\mathbf{S}}^{520}$DDNLEDFDVEEEDEEALIEQR | 7.2 |
| 166 | PRRC2C | ST$\underline{\mathbf{T}}^{2634}$PTSSPFR | 3.2 |
| 167 | PUM2 | GKA$\underline{\mathbf{S}}^{136}$PFEEDQNR | 2.1 |
| 168 | RALBP1 | TGEP$\underline{\mathbf{S}}^{48}$PPHDVLHEPPDTV$\underline{\mathbf{S}}^{62}$DDDKDHGKK | 2.2 |
| 169 | RGS12 | TLPDSQQVPS$\underline{\mathbf{S}}^{850}$PASK | 5.1 |
| 170 | RGS7BP | RG$\underline{\mathbf{S}}^{38}$GSESAHK | 4.9 |
| 171 | RIMS1 | QGSPTQ$\underline{\mathbf{S}}^{1027}$PPADTSFGSR (2xPhospho [$S^{1027}$; S/T]) | 6.5 |
| 172 | RPL6 | KAG$\underline{\mathbf{S}}^{21}$DAAASRPR | 29.4 |
| 173 | RTN3 | SEMCENSEQPQAQPETPTQK (1xPhospho [T]) | 5.2 |
| 174 | SEPTIN7 | $\underline{\mathbf{S}}^{333}$PLAQMoEEER | 2.2 |
| 175 | SGSM1 | RHS$\underline{\mathbf{S}}^{230}$GSMDDRPSISAR (2xPhospho [$S^{230}$; S]) | 5.4 |
| 176 | SH3BP1 | ERTEADLPKPTSPK (1xPhospho [T/S]) | 2.6 |
| 177 | SHANK3 | ST$\underline{\mathbf{S}}^{801}$MQDTVR | 2.1 |
| 178 | SHISA7 | LV$\underline{\mathbf{S}}^{430}$QEHLLLSSPEALR (2xPhospho [$S^{430}$; S])<br>SSSLTPGLGGPDSMoAPR (1xPhospho [S]) | 6.0<br>Tx only |
| 179 | SIPA1L1 | QDPVVHL$\underline{\mathbf{S}}^{1249}$PNK | 2.2 |
| 180 | SKT | AAPTTSSSSSPPSPASPTSLNQGAR (3xPhospho [T/S]) | 2.5 |
| 181 | SLC18A2 | MYTQNNVQPYPVGDDEE$\underline{\mathbf{S}}^{514}$E$\underline{\mathbf{S}}^{516}$D | 2.1 |
| 182 | SLC7A14 | HKQNSEALIANDELDC$\underline{\mathbf{S}}^{769}$PE | 2.2 |
| 183 | SLC9A3R1 | SASSDTSEELNSQDSPKR (3xPhospho [S/T]) | 7.6 |
| 184 | SNX16 | SSSFGSVSTSSTSSK (1xPhospho [T/S]) | 2.1 |
| 185 | SRSF6 | SHSPLPAPPSK (1xPhospho [S]) | 7.2 |

| | | | |
|---|---|---|---|
| 186 | STXBP5 | SRSSSVTSIDKESR (2xPhospho [S]) | 3.3 |
| 187 | STXBP5L | SSSISSIDKDSK (1xPhospho [S]) | 2.1 |
| 188 | TAOK3 | NGPLNE**S**$^{324}$QEEEEDGEQGSNLNR | 2.8 |
| 189 | TEX2 | SL**S**$^{195}$TEVEPK | 2.5 |
| 190 | TMEM245 | SSPS**S**$^{327}$PSPTLGR (2xPhospho [S$^{327}$; S]) | 2.0 |
| 191 | TRIM2 | **S** $^{S440}$PGSGHVK | 64.7 |
| 192 | TRIO | DSDDSAA**T**$^{1824}$PQDETIEER | Tx only |
| 193 | TYRO3 | YIFSPGGLSESPGQLEQQPE**S**$^{859}$PLNENQR (2xPhospho [S$^{859}$; S/Y]) | 3.8 |
| 194 | U2SURP | RH**S**$^{930}$T**S**$^{932}$P**S**$^{934}$PSR | Tx only |
| 195 | UHRF1BP1L | DGSTRLSLDDDGNHNPPSNPVTGK (1xPhospho [S/T]) | Tx only |
| 196 | VPS50 | KK**S**$^{595}$DYSLNK | 8.3 |
| 197 | WDR44 | aceASESDTEEFYDAPEDVHLGTGYPVG**S**$^{27}$PG (3xPhospho [S$^{27}$; S/T]) | 4.0 |
| 198 | ZFR | RRD**S**$^{1054}$DGVDGFEAEGK | 2.8 |
| **Proteins with opposite changes in the phosphopeptide abundance** | | | |
| 199 | CEP170B | GHKHEDGTQSDSEDPLAK (1xPhospho [S/T])<br>GNKHDDG**T**$^{376}$QSDSENAGAHR<br>HEDGTQSDSEDPLAK (1xPhospho [S/T])<br>SVDSRPQPAEHPDHLTITR (1xPhospho [S]) | -4.1<br>-3.3<br>3.9<br>4.5 |
| 200 | DMXL2 | ESEAGTG**S**$^{462}$SEHEDGER (2xPhospho [S$^{462}$; S])<br>MKLDHEL**S**$^{451}$LDR | -4.1<br>3.9 |
| 201 | FAM131B | DTDAY**S** $^{S114}$DL**S**$^{117}$DGEK<br>APTIQPQH**S**$^{102}$HEAVR | Cntr only<br>Tx only |
| 202 | HDGFL3 | FTGYQTIQQQSSSETEGEGGNTADASSEEEGDRVEDGK (1xPhospho [S])<br>EEENK**S**$^{177}$**S**$^{178}$SEGGDAGNDTR | -2.4<br>Tx only |
| 203 | ITSN1 | LPEEPSSEDEQQPEKK (1xPhospho [S])<br>STSIDTGPTESPASLKR (2xPhospho [S/T]) | Cntr only<br>2.3 |
| 204 | KBTBD11 | SGSRPQSPSGDAESR (1xPhospho [S])<br>ASAAEG**S**$^{67}$EA**S**$^{70}$PP**S**$^{73}$LR<br>ASAAEGSEA**S**$^{70}$PPSLR | -2.3<br>4.4<br>10.9 |
| 205 | MAP2 | KSEVQAHSPSRK (1xPhospho [S])<br>GSAQE**S**$^{603}$LDTISPK (2xPhospho [S$^{603}$; S/T]) | -2.0<br>2.2 |
| 206 | MATR3 | DSFDDRGPSLNPVLDYDHGSR (1xPhospho [S])<br>TDAQKTESPAEGK (1xPhospho [T/S]) | Cntr only<br>4.9 |
| 207 | NEFM | SDQAEEGGSEKEG**S**$^{550}$**S**551EKDEGEQEEEEGETEAEGEGEEAEAK<br>(3xPhospho [S$^{550}$; S$^{551}$; S/T])<br>EKAEEEGG**S**$^{715}$EEEGSDR<br>AEEEGGSEEEGSDRSPQESKK (2xPhospho [S])<br>AEEEGG**S** $^{S715}$EEEGSDR<br>AEEEGG**S**$^{715}$EEEG**S**$^{720}$DRSPQESKK (3xPhospho [S$^{715}$; S$^{720}$; S])<br>**S**$^{610}$PVEEVKPKPEAKAGK | Cntr only<br><br>-2.6<br>-2.4<br>-2.3<br>-2.0<br>3.6 |
| 208 | PCLO | DQFGSSH**S**$^{4325}$LPEVQQHMR<br>GAHAHSGPTSAGSSSVP**S**$^{4592}$PGQPG**S**$^{4598}$PSVSK<br>KS**S**$^{1551}$T**S**$^{1553}$FDDDAGR<br>DSFSQES**S**$^{1338}$P**S**$^{1340}$SPSDLAK (3xPhospho [S$^{1338}$; S$^{1340}$; S]) | -3.6<br>2.1<br>2.7<br>5.5 |
| 209 | PRKCE | KLAAGAESPQPASGNSPSEDDR (1xPhospho [S])<br>LAAGAE**S**$^{329}$PQPASGNSPSEDDR (2xPhospho [S$^{329}$; S]) | Cntr only<br>2.3 |
| 210 | SRRM1 | RR**S**$^{561}$P**S**$^{563}$PPPAR<br>R**S**$^{591}$P**T**$^{593}$PPPR<br>RGA**S**$^{723}$A**S**$^{725}$PQGR | -2.1<br>-2.0<br>Tx only |
| 211 | SRRM2 | SGTPPRPGSVTNMQADECTATPQR (3xPhospho [T/S])<br>NNK**S**$^{1465}$V**T**$^{1467}$PQRER | Cntr only<br>4.6 |

**Supplemental Table 2** Phosphopeptides with statistically significant changes in abundance ($P \leq 0.05$) in synaptosomal fractions of EFV-treated (Tx) *vs* control (Cntr) 5XFAD mice from the second treatment paradigm. The identified phosphorylated amino acid residues (STY) are bolded, numbered and underlined. FC, fold change; Ace, acetylated amino acid residue; Mo, oxidized methionine.

| # | Protein | Peptide | FC, Tx/Cntr |
|---|---|---|---|
| **Proteins with a decreased phosphopeptide abundance** | | | |
| 1 | ALDOA | GILAADE**S**$^{90}$TGSIAKR | -8.3 |
| 2 | ALDOART2 | GILAADE**S**$^{36}$TGSIAKR | -8.3 |
| 3 | BASP1 | **S**$^{92}$EGAAEEQPEPAPAPEQEAAAPGPAAGGEAPK | -2.1 |
| 4 | BRAP | EMTDVIIE**T**$^{76}$MK | -5.2 |
| 5 | CANX | **S**$^{553}$DAEEDGVTG**S**$^{563}$QDEEDSKPKAEEDEILNRSPR<br>QKSDAEEDGV**T**$^{561}$GSQDEEDSKPK<br>SDAEEDGVTG**S**$^{563}$QDEEDSKPK | -5.2<br>-4.3<br>-3.7 |
| 6 | GAD1 | TN**S**$^{55}$LEEK | -2.5 |
| 7 | GPM6A | SKEEQELHDIH**S**$^{267}$TR | -4.3 |
| 8 | HEPACAM | LEKQN**S**$^{280}$LEYM$_{O}$DQNDDRLK | -5.5 |
| 9 | MAP1B | ETAAAHQASS**S**$^{1815}$PPIDAATAEPYGFR | -4.8 |
| 10 | PEX5L | NH**S**$^{285}$LEEEFER | -2.3 |
| 11 | PGRMC2 | LLKPGEEPSE**Y**$^{204}$TDEEDTK | -2.3 |
| 12 | PSMD1 | TASAVAGK**T**$^{311}$PDA**S**$^{315}$PEPKDQTLK | -1.8 |
| 13 | RIMS1 | GR**S**$^{346}$QDYPDRLEK | -1.7 |
| 14 | SRCIN1 | AE**S**$^{1044}$EELEVQKPQVK | -2.7 |
| 15 | ZFY1 | SADSSNLK**T**$^{571}$HIK | -14.0 |
| 16 | ZFY2 | SADSSNLK**T**$^{97}$HIK | -14.0 |
| **Proteins with an increased phosphopeptide abundance** | | | |
| 17 | 2010300C02RIK | DM**S**$^{549}$PPEGDVAPPK | 2.5 |
| 18 | AP3D1 | NAEAVK**S**$^{825}$PEKEGVLGVEKK | 3.1 |
| 19 | DPYSL2 | T**S**$^{522}$PAKQQAPPVR | 3.2 |
| 20 | MAP2 | ARVDHGAEIITQ**S**$^{1783}$PSR | 2.2 |
| 21 | VGF | **S**$^{423}$QEEAPGHR | 4.3 |
| **Proteins with opposite changes in the phosphopeptide abundance** | | | |
| 22 | GPRIN1 | ADSA**S**$^{620}$P**S**$^{622}$PRKAESQTSAK<br>ADSA**S**$^{620}$PSPR | -4.0<br>4.1 |
| 23 | MAP1A | AELEEMEEVHP**S**$^{905}$DEEEEETK<br>GKR**S**$^{2841}$PTPGKGPVDR | -5.4<br>2.6 |
| 24 | NEFH | VAPKKEEVK**S**$^{888}$PVK<br>AKPLDVK**S**$^{834}$PEAQTPVQEEAK<br>**S**$^{529}$PGEAKSPAEAK | -5.8<br>3.6<br>5.2 |

**Supplemental Table 3** Phosphopeptides with statistically significant changes in abundance ($P \leq 0.05$) in differentially phosphorylated proteins common in EFV-treated (Tx) *vs* control (Cntr) 5XFAD mice from the first (1TP) and second (2TP) treatment paradigms. The identified phosphorylated amino acid residues (STY) are bolded, numbered and underlined. Peptides in italics are those that overlap in differentially phosphorylated common proteins. FC, fold change; Ace, acetylated amino acid residue; Mo, oxidized methionine.

| **EFV-treated *vs* control 5XFAD mice (1 TP)** | | **EFV-treated *vs* control 5XFAD mice (2 TP)** | |
|---|---|---|---|
| **Peptide** | **FC, Tx/Cntr** | **Peptide** | **FC, Tx/Cntr** |
| **4.1N** | | | |
| $^{67}$DYSEADGL**S**$^{75}$ER<br>$^{481}$ELKPEQET**T**$^{489}$PR<br>*$^{537}$RLPSSPASP**S**$^{546}$PKG**T**$^{550}$PEK* | -7.0<br>-2.7<br>-1.9 | *$^{537}$RLPSSPASPSPKG**T**$^{550}$PEK* | 2.1 |
| **ALDOA** | | | |
| *$^{77}$IVAPGKGILAADES**T**$^{91}$GSIAKR* | -2.0 | *$^{83}$GILAADE**S**$^{90}$TGSIAKR* | -8.3 |
| **BRSK2** | | | |
| $^{413}$SISGASSGLSTSPLSSPR (Phospho [STY]) | 1.2 | $^{439}$G**S**$^{440}$PLP**T**$^{444}$PK | 6.9 |
| **CANX** | | | |
| *$^{553}$SDAEEDGVTG**S**$^{563}$QDEEDSKPK* | -2.3 | *$^{551}$QKSDAEEDGV**T**$^{561}$GSQDEEDSKPK*<br>*$^{553}$SDAEEDGVTG**S**$^{563}$QDEEDSKPK*<br>***S**$^{553}$DAEEDGVTG**S**$^{563}$QDEEDSKPKAEEDEILNRSPR* | -4.5<br>-3.8<br>-5.2 |
| **CEP170B** | | | |
| $^{532}$GASPVTPSTTPPPPTDPQLTK (Phospho [STY]) | -1.8 | $^{351}$GHKHEDGTQSDSEDPLAK (1xPhospho [T/S])<br>$^{354}$HEDGTQSDSEDPLAK (1xPhospho [T/S])<br>$^{369}$GNKHDDG**T**$^{376}$QSDSENAGAHR<br>$^{1384}$SVDSRPQPAEHPDHLTITR (1xPhospho [S]) | -4.1<br>3.9<br>-3.3<br>4.5 |
| **CTNND2** | | | |
| $^{262}$GG**S**$^{264}$PLTTTQGG**S**$^{273}$PTKLQR<br>$^{452}$TSTAP**S**$^{457}$SPGVDSVPLQR<br>$^{529}$SP**S**$^{531}$ID**S**$^{534}$IQKDPR | -2.6<br>-2.3<br>Tx only | **T**$^{1078}$PSI**S**$^{1082}$PVR | 4.2 |
| **DPYSL2** | | | |
| *$^{512}$TVTPA**S**$^{517}$**S**$^{518}$AKT**S**$^{522}$PAKQQAPPVR* | | *$^{497}$GLYDGPVCEV**S**$^{507}$V**T**$^{509}$PK*<br>*$^{521}$T**S**$^{522}$PAKQQAPPVR* | -1.3<br>3.2 |
| **EIF4B** | | | |
| $^{494}$SQSSDTEQPSPTSGGGKVAAVQPPEEGPSR (2xPhospho [STY]) | -1.8 | $^{201}$ARPTTDSFDDYPPR (1xPhospho [S/T]) | 3.3 |
| **FAM131B** | | | |
| $^{312}$VSDVTSSGVQSFDEEEGDANN (Phospho [STY]) | -2.3 | $^{94}$APTIQPQH**S**$^{102}$HEAVR<br>$^{109}$DTDAY**S**$^{114}$DL**S**$^{117}$DGEK | Tx only<br>Cntr only |
| **FMN2** | | | |
| $^{483}$GATADDSGGGSPVLAAK (Phospho [STY]) | -1.8 | **S**$^{720}$IQT**S**$^{724}$PTEEGR | 2.2 |
| **FXYD7** | | | |
| $^{65}$SCKSELP**S**$^{72}$SAPGGGGV | Tx only | $^{54}$ADSRSESPTCK (2xPhospho [S]) | Tx only |
| **GAP43** | | | |
| $^{182}$AAQPPTE**T**$^{189}$AES**S**$^{193}$QAEEEK | -4.4 | $^{126}$AGSAETESAAKATTDNSPSSK (1xPhospho [S]) | 4.8 |
| **HECW1** | | | |

| | | | |
|---|---|---|---|
| $^{1057}$SY**S**$^{1059}$AGEASEVSR | -6.7 | $^{577}$GSTTEEEDGLEEESTLK (1xPhospho [S/T]) | Cntr only |
| **MAP1B** | | | |
| $^{988}$ESVV**S**$^{992}$GDDR | -3.3 | $^{1805}$ETAAAHQASS**S**$^{1815}$PPIDAATAEPYGFR<br>$^{2193}$HMDPPPAPMQDR**S**$^{2205}$P**S**$^{2207}$PR | -4.5<br>6.0 |
| **MAP2** | | | |
| $^{1592}$SGTSTPTTPGSTAITPGTPPSYSSR (Phospho [STY]) | -1.9 | $^{598}$GSAQE**S**$^{603}$LDTISPK<br>$^{1410}$ARVDHGAEIITQ**S**$^{422}$PSR<br>$^{1478}$KSEVQAHSPSRK (1xPhospho [S]) | 2.2<br>2.2<br>-2.1 |
| **MAP7D1** | | | |
| $^{114}$SSQPSPTTVPA**S**$^{125}$DSPPAKQDVK | -3.7 | $^{533}$AAEEKEPAAPA**S**$^{544}$PAPSPVPSPTPAQPQK | 3.7 |
| **NEFH** | | | |
| $^{727}$SPAEAK **S**$^{733}$PAAVKSPGEAK | -7.3 | **S**$^{529}$PGEAKSPAEAK<br>**S**$^{571}$PAEAK **S**$^{577}$PAEPK<br>$^{827}$AKPLDVK**S**$^{834}$PEAQTPVQEEAK<br>$^{879}$VAPKKEEVK **S**$^{888}$PVK | 5.2<br>Tx only<br>3.6<br>-5.7 |
| **NOP58** | | | |
| *$^{505}$EEPL**S**$^{509}$EEEPCTSTAVP**S**$^{521}$PEK* | 5.4 | *$^{502}$HIKEEPL**S**$^{509}$EEEPCTSTAVPSPEKK* | -7.6 |
| **PALM** | | | |
| $^{110}$ENSAAPSPGRPQ**S**$^{122}$A**S**$^{124}$PAK | -2.4 | $^{151}$TSTPVRSPGGSTMMK (2xPhospho [S/T]) | 2.1 |
| **PCLO** | | | |
| $^{200}$SEGITKPSLQQP**S**$^{212}$PK<br>$^{1396}$DI**S**$^{1398}$ISEEEIK | -1.9<br>-8.5 | $^{1331}$DSFSQES**S**$^{1338}$P**S**$^{1340}$SPSDLAK<br>$^{1549}$KS**S**$^{1551}$T**S**$^{1553}$FDDDAGR<br>$^{4318}$DQFGSSH**S**$^{4325}$LPEVQQHMR<br>$^{4575}$GAHAHSGPTSAGSSSVP**S**$^{4592}$PGQPG**S**$^{4598}$PSVSK | 5.5<br>2.6<br>-3.6<br>2.1 |
| **PHLDB1** | | | |
| $^{439}$TLQPPE**S**$^{445}$PR | -4.2 | **S**$^{520}$PS$^{522}$PTLGESLAPR | Cntr only |
| **PRKCE** | | | |
| *$^{321}$KKLAAGAE**S**$^{329}$PQPASGNSP**S**$^{339}$EDDR* | -3.0 | *$^{322}$KLAAGAESPQPASGNSPSEDDR* (1xPhospho [S])<br>*$^{323}$LAAGAE**S**$^{329}$PQPASGNSPSEDDR* | Cntr only<br>2.2 |
| **RGS12** | | | |
| $^{879}$SLNEDVGEEDSEK (Phospho [STY]) | -3.2 | $^{840}$TLPDSQQVPS**S**$^{850}$PASK | 5.1 |
| **RIMS1** | | | |
| $^{1116}$SCDNASAK**S**$^{1124}$**S**$^{1125}$DSDVSDVSAISR | -8.0 | $^{344}$GR**S**$^{346}$QDYPDRLEK<br>$^{1021}$QGSPTQ**S**$^{1027}$PPADTSFGSR | -1.7<br>6.5 |
| **RTN3** | | | |
| $^{2}$AceAESSAATQSPSVSSSSSGAEPSALGGGGGSPGACPALGAK (2xPhospho [STY]) | -2.0 | $^{509}$SEMCENSEQPQAQPETPTQK (1xPhospho [T]) | 5.2 |
| **SHISA7** | | | |
| *$^{248}$SSSLTPGLGGPDSMoAPR* (Phospho [STY]) | -1.9 | *$^{248}$SSSLTPGLGGPDSMoAPR* (1xPhospho [S])<br>$^{428}$LVSQEHLLLSSPEALR (2xPhospho [S]) | Tx only<br>6.0 |
| **SKT** | | | |
| $^{1029}$LELSEDSPNSEQELDK (Phospho [STY]) | -1.9 | $^{1890}$AAPTTSSSSSPPSPASPTSLNQGAR (3xPhospho [S/T]) | 2.5 |
| **SRCIN1** | | | |
| $^{544}$DSGSS**S**$^{549}$VFAESPGGK<br>$^{544}$DSGSS**S**$^{549}$VFAESPGGKAR<br>$^{655}$S**S**$^{654}$GATPVSGPPPPSASSTPAGQPTAVSR | -15.3<br>-3.1<br>-4.5 | $^{1042}$AE**S**$^{1044}$EELEVQKPQVK | -2.7 |

| | | | |
|---|---|---|---|
| $^{655}$S**<u>S</u>**$^{\mathbf{654}}$GA**<u>T</u>**$^{\mathbf{657}}$PVSGPPPPSASSTPAGQPT AVSR | -10.3 | | |

**Supplemental Table 4** Phosphopeptides with statistically significant changes in abundance ($P \leq 0.05$) in differentially phosphorylated proteins common in *Cyp46a1*$^{-/-}$ (KO) *vs* wild type (WT) mice and EFV-treated 5XFAD (Tx) *vs* control (Cntr) 5XFAD mice from the second treatment paradigm (2TP). The identified phosphorylated amino acid residues (STY) are bolded, numbered and underlined. Peptides in italics are those that overlap in differentially phosphorylated common proteins. FC, fold change; Mo, oxidized methionine.

| ***Cyp46a1*$^{-/-}$ *vs* wild type mice** | | **EFV-treated *vs* control 5XFAD mice (2 TP)** | |
|---|---|---|---|
| **Peptide** | **FC, KO/WT** | **Peptide** | **FC, Tx/Cntr** |
| **2010300C02RIK** | | | |
| $^{959}$TR**S$^{961}$**PEQPGTKPPLPR | 1.6 | $^{547}$DM**S$^{549}$**PPEGDVAPPK | 2.5 |
| **ADCY9** | | | |
| $^{605}$GQGTA**S$^{610}$**PG**S$^{613}$**VSDLAQTVK | -4.7 | $^{1291}$ASLG**S$^{1295}$**DDSTQAK | Cntr only |
| $^{1327}$AIEKD**S$^{1332}$**CEDIGVEEASELSK | -2.2 | | |
| **ANK2** | | | |
| $^{1697}$KG**S$^{1699}$S$^{1700}$**EE**S$^{1703}$**VDEDRGLVPEPLPTAK | -1.5 | $^{2821}$EIASPSSPVK (2xPhospho [S]) | 3.7 |
| $^{2350}$TAEGTEPKPQGAIR**S$^{2364}$**PQGLELPLPNR | -3.3 | | |
| $^{3682}$TEGD**S$^{3686}$**PAAAL**S$^{3692}$**PQMHQEPVQQDFSGK | -9.9 | | |
| **BSN** | | | |
| $^{987}$GEHSSTLPASTPSYTSGTSPTSLSSLEEDSDS**S$^{1019}$**PSR<br>$^{1099}$SKTPPSNL**S$^{1108}$**PIEDA**S$^{1114}$**PTEELR | -12.7<br>-7.1 | $^{1039}$SHGPLLPTIEDSSEEEELREEEELLREQEK (1xPhospho [S])<br>$^{1233}$SQGSFEYQDTQDHDYGGR (1xPhospho [S])<br>$^{3156}$QT**S$^{3158}$**LADLEQK | 10.8<br>2.2<br>Tx only |
| **CEP170B** | | | |
| $^{1347}$SP**S$^{1349}$**LGNVPNTPASTISAR | 1.8 | $^{351}$GHKHEDGTQSDSEDPLAK (1xPhospho [T/S])<br>$^{354}$HEDGTQSDSEDPLAK (1xPhospho [T/S])<br>$^{369}$GNKHDDG**T$^{376}$**QSDSENAGAHR<br>$^{1384}$SVDSRPQPAEHPDHLTITR (1xPhospho [S]) | -4.1<br>3.9<br>-3.3<br>4.5 |
| **CHGB** | | | |
| $^{131}$EGVDDQESLRPSNQQA**S$^{147}$**K | 1.9 | $^{186}$HIED**S$^{190}$**GEKPNTFSNK | -2.4 |
| **CTNND2** | | | |
| $^{28}$NSSL**S$^{32}$**PGLNTSNGDGSETETTSAILASVK | -1.9 | **T$^{1078}$**PSI**S$^{1082}$**PVR | 4.2 |
| **DBN1** | | | |
| $^{141}$LS**S$^{143}$**PVLHR | -38.1 | **S$^{385}$**P**S$^{387}$**DSSTASTPIAEQIER | Cntr only |
| **DMXL2** | | | |
| $^{2707}$S**S$^{2708}$**DDIDYR | -2.2 | $^{444}$MKLDHEL**S$^{451}$**LDR<br>$^{455}$ESEAGTG**S$^{462}$**SEHEDGER | 3.9<br>-4.1 |
| **DNAJC6** | | | |
| $^{588}$TATSASA**S$^{595}$**PTLR | -3.1 | $^{33}$GASSPDMoEPSYGGGLFDMVK, (1xPhospho [S]) | -5.3 |
| | | $^{466}$CEEDHAALVNQE**S$^{478}$**EQ**S$^{481}$**DDELLTLSSPHGNAEGDKPHGAK | -2.1 |
| **DPYSL2** | | | |
| *$^{497}$GLYDGPVCEVSV**T$^{509}$**PK**T$^{512}$**V**T$^{514}$**PASSAK* | -2.1 | *$^{497}$GLYDGPVCEV**S$^{507}$**V**T$^{509}$**PK* | -1.3 |

| | | | |
|---|---|---|---|
| | | *$^{521}$T**S**$^{522}$PAKQQAPPVR* | 3.2 |
| **EIF4B** | | | |
| $^{593}$YAALSVDGEDEDEGDDC**T**$^{610}$E | -4.2 | $^{201}$ARPTTDSFDDYPPR<br>(1xPhospho [S/T]) | 3.3 |
| **GAP43** | | | |
| $^{82}$EGDGSATTDAAPAT**S**$^{96}$PK | -2.6 | $^{126}$AGSAETESAAKATTDNSPSSK<br>(1xPhospho [S]) | 4.8 |
| **GPR158** | | | |
| $^{75}$V**S**$^{76}$LPTANPDVSSGITQIK | -1.2 | $^{237}$RG**S**$^{239}$NQGPR | 49.5 |
| **HDGF** | | | |
| $^{158}$AGDVLED**S**$^{165}$PKRPK | -7.8 | $^{127}$GSAEGSSDEEGKLVIDEPAKEK<br>(1xPhospho [S]) | Cntr only |
| **KBTBD11** | | | |
| *$^{61}$ASAAEGSEA**S**$^{70}$PP**S**$^{73}$LR* | -2.9 | *$^{61}$ASAAEGSEA**S**$^{70}$PPSLR*<br>*$^{61}$ASAAEG**S**$^{67}$EA**S**$^{70}$PP**S**$^{73}$LR*<br>$^{314}$SGSRPQSPSGDAESR (1xPhospho [S]) | 10.9<br>4.4<br>-2.3 |
| **MACF1** | | | |
| $^{4474}$AFLAELEQN**S**$^{4483}$PK | -3.2 | $^{3887}$QG**S**$^{3889}$FSEDVISHK | Tx only |
| **MAP1B** | | | |
| $^{923}$FEDEGAGFEES**S**$^{934}$ETGDYEEK<br>$^{987}$RE**S**$^{989}$VVS$^{992}$GDDRAEEDMoDDVLEK<br>$^{1008}$GEAEQ**S**$^{1013}$EEEGEEEDKAEDAR<br>$^{1195}$DYNASAS**T**$^{1202}$ISPPSSMoEEDKFSK<br>$^{1195}$DYNASASTI**S**$^{1204}$PPSSMoEEDK<br>$^{1195}$DYNASASTI**S**$^{1204}$PP**S**$^{1207}$SMEEDKFSK<br>$^{1240}$DVSDERL**S**$^{1247}$PAK**S**$^{1251}$P**S**$^{1253}$LSPSPPS PIEK<br>$^{1389}$VLSPLR**S**$^{1395}$PPLLGSESPYEDFLSADS K<br>$^{1766}$VQSLEGEKL**S**$^{1775}$PK**S**$^{1778}$DI**S**$^{1781}$PLTPR<br>**T**$^{1945}$PEEGGYSYEISEK | 2.6<br>2.1<br>2.7<br>2.6<br>10.0<br>-5.9<br>-3.0<br>KO only<br>-1.6<br>1.3 | $^{1805}$ETAAAHQASS**S**$^{1815}$PPIDAATAEPYGFR<br>$^{2193}$HMDPPPAPMQDR**S**$^{2205}$P**S**$^{2207}$PR | -4.5<br>6.0 |
| **MAP2** | | | |
| $^{1020}$GLS**S**$^{1023}$VPEVAEVEPTTK<br>$^{1530}$DKVTDGISK**S**$^{1539}$PEKR | -2.8<br>-7.0 | $^{598}$GSAQE**S**$^{603}$LDTISPK<br>$^{1410}$ARVDHGAEIITQ**S**$^{422}$PSR<br>$^{1478}$KSEVQAHSPSRK (1xPhospho [S]) | 2.2<br>2.2<br>-2.1 |
| **MAPT** | | | |
| **S**$^{57}$TPTAEDVTAPLVDER<br>$^{467}$TTP**S**$^{470}$PKTPPG**S**$^{477}$GEPPK<br>$^{676}$AKTDHGAEIVYK**S**$^{688}$PVV**S**$^{692}$GDT**S**$^{696}$PR<br>$^{688}$SPVV**S**$^{692}$GDT**S**$^{696}$PR<br>$^{688}$SPVVSGDT**S**$^{696}$PR | -1.9<br>-1.3<br>-1.7<br>-2.5<br>-5.0 | $^{646}$IGS$^{648}$LDNITHVPGGGNKK | Cntr only |
| **NEFH** | | | |
| **S**$^{757}$PAEAK **S**$^{763}$PIEVK<br>**S**$^{757}$PAEAK **S**$^{763}$PIEVK**S**$^{769}$PEK<br>$^{793}$AK**S**$^{795}$PVKEDIKPPAEAK**S**$^{809}$PEK | 1.2<br>1.5<br>1.7 | **S**$^{529}$PGEAKSPAEAK<br>**S**$^{571}$PAEAK **S**$^{577}$PAEPK<br>$^{827}$AKPLDVK**S**$^{834}$PEAQTPVQEEAK<br>$^{879}$VAPKKEEVK **S**$^{888}$PVK | 5.2<br>Tx only<br>3.6<br>-5.7 |
| **NEFM** | | | |
| $^{27}$VSG**S**$^{30}$PSSGFR<br>$^{488}$EEAEEKEEEPEAEK**S**$^{502}$PVK**S**$^{506}$PEAK | -3.8<br>2.1 | $^{537}$SDQAEEGGSEKEG**S**$^{550}$**S**$^{551}$EKDEGEQEEE EGETEAEGEGEEAEAK<br>**S**$^{610}$PVEEVKPKPEAKAGK<br>$^{707}$EKAEEEGGS$^{715}$EEEGSDR<br>$^{709}$AEEEGG**S**$^{715}$EEEGSDR<br>$^{709}$AEEEGG**S**$^{715}$EEEG**S**$^{720}$DRSPQESKK | Cntr only<br>3.6<br>-2.6<br>-2.3<br>-2.0 |

| | | | |
|---|---|---|---|
| **PPP1R7** | | | |
| $^{35}$HGGGGIVANL**S**$^{\mathbf{45}}$EQ**S**$^{\mathbf{48}}$LKDGVDR | -1.9 | $^{21}$RVESEESGDEEGKK (1xPhospho [S]) | -2.4 |
| **RALBP1** | | | |
| $^{82}$TEGYAAFQED**S**$^{\mathbf{92}}$**S**$^{\mathbf{93}}$GDEAESPSK | 1.7 | $^{44}$TGEP**S**$^{\mathbf{48}}$PPHDVLHEPPDTV**S**$^{\mathbf{62}}$DDDKDHG KK | 2.2 |
| **SERINC1** | | | |
| *$^{344}$LTLT**S**$^{\mathbf{348}}$DESTLIEDGNGR* | -3.0 | *$^{344}$LTLTSDESTLIEDGNGR* (2xPhospho [S/T]) | -2.3 |
| **SHANK3** | | | |
| **S**$^{\mathbf{1510}}$LGEEPVGGLGSLLDPAK | -1.9 | $^{799}$ST**S**$^{\mathbf{801}}$MQDTVR | 2.0 |
| **SYT1** | | | |
| *$^{120}$DDDAETGL**T**$^{\mathbf{128}}$DGEEKEEPKEEEK* | 1.4 | *$^{120}$DDDAETGL**T**$^{\mathbf{128}}$DGEEKEEPKEEEK* | -16.0 |
| **THRAP3** | | | |
| $^{676}$IDI**S**$^{\mathbf{679}}$PSTFR | -1.6 | $^{53}$SYSPAHNR, (2xPhospho [S/Y])<br>$^{182}$SSSKDSRPSQAAGDNQGDEAK (1xPhospho [S])<br>$^{686}$HGLTHEELK**S**$^{\mathbf{695}}$PR | -2.6<br>-3.2<br>Cntr only |
| **TOP2B** | | | |
| $^{1503}$IVETINSD**S**$^{\mathbf{1511}}$DSEFGIPK | -1.8 | $^{1444}$SEDDSAKFDSNEEDTASVFAPSFGLK (1xPhospho [S/T])<br>$^{1451}$FDSNEEDTASVFAPSFGLK (1xPhospho [S/T]) | -2.5<br>-3.0 |

**Supplemental Table 5** Phosphopeptides with statistically significant changes in abundance ($P \leq 0.05$) in differentially phosphorylated proteins common in *Cyp46a1*$^{-/-}$ (KO) *vs* wild type (WT) mice and EFV-treated 5XFAD (Tx) *vs* control (Cntr) 5XFAD mice from the first (1TP) and (2TP) second treatment paradigms. The identified phosphorylated amino acid residues (STY) are bolded, numbered and underlined. Peptides in italics are those that overlap in differentially phosphorylated common proteins. FC, fold change; Mo, oxidized methionine.

| ***Cyp46a1*$^{-/-}$ *vs* wild type mice** | | **EFV-treated *vs* control 5XFAD mice (1 TP)** | | **EFV-treated *vs* control 5XFAD mice (2 TP)** | |
|---|---|---|---|---|---|
| **Peptide** | **FC, KO/WT** | **Peptide** | **FC, Tx/Cntr** | **Peptide** | **FC, Tx/Cntr** |
| **CEP170B** | | | | | |
| $^{1347}$SP**S$^{1349}$**LGNVPNTPASTISAR | 1.8 | $^{532}$GASPVTPSTTPPPPTDPQLTK (Phospho [STY]) | -1.8 | $^{351}$GHKHEDGTQSDSEDPLAK (1xPhospho [T/S])<br>$^{354}$HEDGTQSDSEDPLAK (1xPhospho [T/S])<br>$^{369}$GNKHDDG**T$^{376}$**QSDSENAGAHR<br>$^{1384}$SVDSRPQPAEHPDHLTITR (1xPhospho [S]) | -4.1<br>3.9<br>-3.3<br>4.5 |
| **CTNND2** | | | | | |
| $^{28}$NSSL**S$^{32}$**PGLNTSNGDGSETETTSAILASVK | -1.9 | $^{262}$GG**S$^{264}$**PLTTTQGG**S$^{273}$**PTKLQR<br>$^{452}$TSTAP**S$^{457}$**SPGVDSVPLQR<br>$^{529}$SP**S$^{531}$**ID**S$^{534}$**IQKDPR | -2.6<br>-2.3<br>Tx only | **T$^{1078}$**PSI**S$^{1082}$**PVR | 4.2 |
| **DPYSL2** | | | | | |
| *$^{497}$GLYDGPVCEVSV**T$^{509}$**PK**T$^{512}$**V**T$^{514}$**PASSAK* | -2.1 | *$^{512}$TVTPA**S$^{517}$S$^{518}$**AKT**S$^{522}$**PAKQQAPPVR* | -2.2 | *$^{497}$GLYDGPVCEV**S$^{507}$**V**T$^{509}$**PK*<br>*$^{521}$T**S$^{522}$**PAKQQAPPVR* | -1.3<br>3.2 |
| **EIF4B** | | | | | |
| $^{593}$YAALSVDGEDEDEGDDC**T$^{610}$**E | -4.2 | $^{494}$SQSSDTEQPSPTSGGGKVAAVQPPEEGPSR (2xPhospho [STY]) | -1.8 | $^{201}$ARPTTDSFDDYPPR (1xPhospho [S/T]) | 3.3 |
| **GAP43** | | | | | |
| $^{82}$EGDGSATTDAAPAT**S$^{96}$**PK | -2.6 | $^{182}$AAQPPTE**T$^{189}$**AES**S$^{193}$**QAEEEK | -4.4 | $^{126}$AGSAETESAAKATTDNSPSSK (1xPhospho [S]) | 4.8 |
| **MAP1B** | | | | | |
| $^{923}$FEDEGAGFEES**S$^{934}$**ETGDYEEK<br>*$^{987}$RE**S$^{989}$**VVS$^{992}$GDDRAEEDMoDDVLEK*<br>$^{1008}$GEAEQ**S$^{1013}$**EEEGEEEDKAEDAR<br>$^{1195}$DYNASAS**T$^{1202}$**ISPPSSMoEEDKFSK<br>$^{1195}$DYNASASTI**S$^{1204}$**PPSSMoEEDK<br>$^{1195}$DYNASASTI**S$^{1204}$**PP**S$^{1207}$**SMEEDKFSK<br>$^{1240}$DVSDERL**S$^{1247}$**PAK**S$^{1251}$**P**S$^{1253}$**LSPSPPSPIEK | 2.6<br>2.1<br>2.7<br>2.6<br>10.0<br>-5.9<br>-3.0 | *$^{988}$ESVV**S$^{992}$**GDDR* | -3.3 | $^{1805}$ETAAAHQASS**S$^{1815}$**PPIDAATAEPYGFR<br>$^{2193}$HMDPPPAPMQDR**S$^{2205}$**P**S$^{2207}$**PR | -4.5<br>6.0 |

| | | | | | |
|---|---|---|---|---|---|
| $^{1389}$VLSPLR**S**$^{1395}$PPLLGSESPYEDFLSADSK | KO only | | | | |
| $^{1766}$VQSLEGEKL**S**$^{1775}$PK**S**$^{1778}$DI**S**$^{1781}$PLTPR | -1.6 | | | | |
| **T**$^{1945}$PEEGGYSYEISEK | 1.3 | | | | |
| **MAP2** | | | | | |
| $^{1020}$GLS**S**$^{1023}$VPEVAEVEPTTK | -2.8 | $^{1592}$SGTSTPTTPGSTAITPGTPPSYSSR (Phospho [STY]) | -1.9 | $^{598}$GSAQE**S**$^{603}$LDTISPK | 2.2 |
| $^{1530}$DKVTDGISK**S**$^{1539}$PEKR | -7.0 | | | $^{1410}$ARVDHGAEIITQ**S**$^{422}$PSR | 2.2 |
| | | | | $^{1478}$KSEVQAHSPSRK (1xPhospho [S]) | -2.1 |
| **NEFH** | | | | | |
| **S**$^{757}$PAEAK **S**$^{763}$PIEVK | 1.2 | $^{727}$SPAEAK **S**$^{733}$PAAVKSPGEAK | -7.3 | **S**$^{529}$PGEAKSPAEAK | 5.2 |
| **S**$^{757}$PAEAK **S**$^{763}$PIEVK**S**$^{769}$PEK | 1.5 | | | **S**$^{571}$PAEAK **S**$^{577}$PAEPK | Tx only |
| $^{793}$AK**S**$^{795}$PVKEDIKPPAEAK**S**$^{809}$PEK | 1.7 | | | $^{827}$AKPLDVK**S**$^{834}$PEAQTPVQEEAK | 3.6 |
| | | | | $^{879}$VAPKKEEVK **S**$^{888}$PVK | -5.7 |
